# From networks of protein interactions to networks of functional dependencies

**DOI:** 10.1186/1752-0509-6-44

**Published:** 2012-05-20

**Authors:** Davide Luciani, Gianfranco Bazzoni

**Affiliations:** 1 Unit of Clinical Knowledge Engineering, Mario Negri Institute of Pharmacological Research, Milan, I-20156, Italy; 2 Laboratory of Systems Biology, Mario Negri Institute of Pharmacological Research, Milan, I-20156, Italy

**Keywords:** Protein interaction networks, Biological functions, Markov representations, Peroxisomes, Cell budding, Polarized growth, *Saccharomyces cerevisiae*

## Abstract

**Background:**

As protein-protein interactions connect proteins that participate in either the same or different functions, networks of interacting and functionally annotated proteins can be converted into process graphs of inter-dependent function nodes (each node corresponding to interacting proteins with the same functional annotation). However, as proteins have multiple annotations, the process graph is non-redundant, if only proteins participating directly in a given function are included in the related function node.

**Results:**

Reasoning that topological features (e.g., clusters of highly inter-connected proteins) might help approaching structured and non-redundant understanding of molecular function, an algorithm was developed that prioritizes inclusion of proteins into the function nodes that best overlap protein clusters. Specifically, the algorithm identifies function nodes (and their mutual relations), based on the topological analysis of a protein interaction network, which can be related to various biological domains, such as cellular components (e.g., peroxisome and cellular bud) or biological processes (e.g., cell budding) of the model organism *S. cerevisiae*.

**Conclusions:**

The method we have described allows converting a protein interaction network into a non-redundant process graph of inter-dependent function nodes. The examples we have described show that the resulting graph allows researchers to formulate testable hypotheses about dependencies among functions and the underlying mechanisms.

## Background

In recent years, small- and large-scale experiments have produced a considerable wealth of information about the physical interactions of thousands of molecules. Proteins, in particular, have been reported to interact physically with other proteins, as well as with genes, transcripts and metabolites. Various types of protein-protein interactions (PPI) have been documented, ranging from PPI that bring about assembly of stable protein complexes to PPI that cause transient modifications (e.g., phosphorylation) of target proteins. Retrieving PPI from available databases enables system-level analysis of protein interactomes in various model organisms. Furthermore, suitable tools are available for representing the interactomes, including the PPI networks, which display proteins and PPI as nodes and edges, respectively [1].

In addition to the interactions, also the functions of numerous proteins have been characterized broadly. Evidence about protein function can be retrieved (among other sources) from the vocabularies of Gene Ontology (GO), which annotate each protein with its contribution to biological processes, localization to cellular components and performance of molecular functions. Each GO vocabulary is hierarchically structured according to ontological relations among the annotations, with the terms ‘biological process’, ‘cellular component’ and ‘molecular function’ being the roots of each graph [2].

To find out which functions are associated with the proteins of a PPI network, a common approach is gene annotation enrichment analysis, which identifies the functional annotations that are significantly more frequent in the PPI network than in a reference set of proteins [3]. However, it is not the purpose of traditional enrichment analysis either to consider the interactions among proteins or to define the relationships among biological functions. Yet, this information is essential to address issues that are better analyzed in functional than molecular terms (e.g., mechanisms of embryonic development or manifestations of inherited diseases). Ideally, a graph that represents functions and their hypothetical causal relations (as nodes and edges, respectively) would be useful in designing experiments aimed at testing whether the manipulation of a function (including, but not limited to, the manipulation of its protein components) affects other functions.

Thus, we devised a method to elaborate PPI networks towards a functional synthesis that might be regarded to as one of the typical goals of systems biology. Actually, mapping relationships among the functions that annotate physically interacting proteins is not an unprecedented attempt [4,5]. Nevertheless, current algorithms for mining biochemical data are not designed to distinguish direct functional relations from functional relations that are mediated by other functions. In addition, they are not designed to control the level of details of the final representation, which greatly impacts on the represented steps through which a functional relation is obtained.

On the other hand, no systematic analysis has been performed so far, to arrive at a graphical representation of relationships among functions that takes into account the inherent limitations of the evidence exploited. First and foremost, GO annotations refer to functions that are impacted by the manipulation of a gene or a gene product (for instance, the annotations inferred from mutant phenotypes or direct assays, respectively), without referring to how direct the impact is. Second, annotations are heterogeneous with respect to the method, which can be based on experimental evidence, computational inference or author statement. Third, also the experimental assays used for discovering PPI are dissimilar, as ‘binary’ assays (e.g., yeast two-hybrid) report *bona fide* direct PPI, whereas ‘cluster’ assays (e.g., affinity capture assays) establish the existence of PPI among all the proteins that belong to the same complex [6]. Finally, edges in the PPI networks are often assumed as transitive (i.e., if protein A influences protein B and B influences protein C, then A does also influence C). While the assumption is essential to interpret the PPI network as an ordered layout of interacting molecules, it does not exclude the possibility that different copies of the same protein might engage in different PPI.

Thus, starting from a PPI network, our goal is to elaborate a graph *G* = (V, E), herein called *process graph* (PG), where V is a set of *function nodes* (FN) that indicate biological functions and E is a set of edges that portray relations among functions. As long as information about the direction of the relations is not available, the relations cannot be characterized fully as ‘causal’, leaving *G* as an undirected graph rather than a more easily interpretable Directed Acyclic Graph (DAG) [7]. Nevertheless, dependencies can be read off from the whole ensemble of edges even in undirected graphs [8]. For instance, suppose that observing A is irrelevant to make an inference about C, if B is observed. In this case, to represent the transitivity of these dependencies, A should be graphically linked to B and B to C, but A should not be linked to C. In general, to signify that the presence of a variable makes it irrelevant to observe another variable, a graph must comply with the *markov property*: all information about a phenomenon is contained in the impact of its adjacent neighbours in the graph [8,9]. Hereafter, we provide the rational basis for automatically inferring such PG representation from the topological information that is included in the PPI network.

Given that FN are defined as annotations of proteins and that relationships among proteins are assumed to be transitive, one way to make the PG comply with the markov property is to generate FN that are defined by distinct subsets of proteins without overlaps. However, as anticipated, the individual proteins are often annotated with several functions, which makes frequent the occurrence of several functions covering the same set of proteins. To tackle the issue, it was further assumed that a protein the more likely belongs to a FN, the more interactions it has with the other proteins of the FN [10]. On these grounds, topological analysis was applied to discriminate between proteins supporting a function directly and proteins acting through a path of intermediate functions. This way, by approaching the goal of the markov property, isolated findings can be understood within a coherent representation of the whole biological phenomenon under scrutiny.

To test our method, we have exploited available knowledge about protein interactions and annotations. As domain of biological interest, we first focused on a cellular component (i.e., the peroxisome) of the eukaryotic model organism *Saccharomyces cerevisiae*, selected its annotated proteins together with the corresponding PPI network and applied our algorithm to define the relevant PG of peroxisomal functions. Then, to focus on two additional domains, i.e., another cellular component and a biological process, we have applied a similar procedure to other PPI networks composed of yeast proteins that localize to the cellular bud and that contribute to cell budding, respectively. The choice of well known domains offers the opportunity to exploit additional information about the directionality of causal relations, so that the undirected edges of the PG can be turned into directed edges and be more easily confirmed by the current biological knowledge. We argue that a successful validation of the PG obtained from well known domains would make a PG obtained from less known domains particularly useful in defining a restricted class of markov equivalent DAG [11]. In turn, each DAG would correspond to an experimentally testable hypothesis representing a fully causal explanation of the investigated domain [7].

## Results

### The protein-protein interaction network of the *S. cerevisiae* peroxisome

To assemble the PPI network of the *S. cerevisiae* peroxisome, we identified first the peroxisomal core proteins and then their mutual PPI. In addition, the PPI network was extended to include the first-degree neighbors (i.e., the non-core proteins that are linked to at least one core protein by means of a PPI). All the proteins of the yeast peroxisome network are listed in Additional file 1, while the PPI network is shown in Additional file 2.

The network consists of 450 proteins (61 core and 389 neighbor proteins) and 2,433 PPI (128 core-core, 501 core-neighbor and 1,804 neighbor-neighbor PPI), which have been detected by binary or cluster assays (Table 1). The network has average connectivity of 10.8, average clustering coefficient of 0.20 and characteristic path length of 2.8. Together with the protein annotations in GO, the PPI network is the starting point to assemble the PG of the yeast peroxisome.

**Table 1:** Types of assays used to detect the PPI reported in the PPI network of the yeast peroxisome

**Assay**	**PPI**	**%**
***Binary methods***		
Two-hybrid	546	22,2
Biochemical Activity	295	12,0
PCA	194	7,9
	1,035	42,1
***Cluster methods***		
Affinity Capture-MS	1,112	45,3
Affinity Capture-Western	164	6,7
Co-crystal Structure	1	0,0
Co-fractionation	62	2,5
Co-localization	9	0,4
Co-purification	17	0,7
Far Western	2	0,1
Reconstituted Complex	55	2,2
	1,422	57,9
***Total Binary and Cluster***	2,457	100

For each assay, the PPI detections are shown as total number and percentage. The total number of PPI detections exceeds the actual number of PPI (2,457 versus 2,433), because 24 PPI were detected with two methods. See also Additional file 1 (for a list of the proteins in the PPI network) and Additional file 2 (for a visual display of the PPI network).

### From protein interactions and annotations to the function nodes

**Figure 1:**
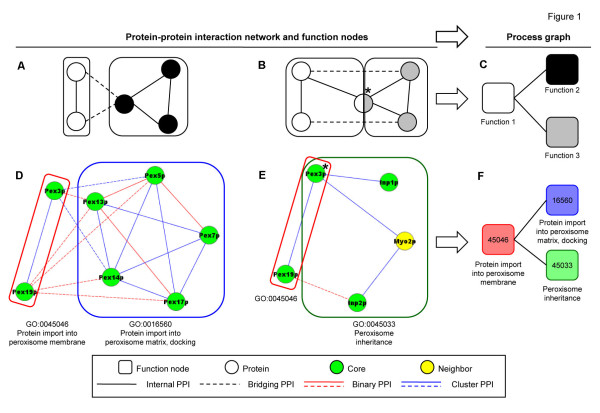
**From the protein-protein interaction network to the function nodes and the process graph.** (**A, D**) When two (or more) interacting proteins (*circles*) within a PPI network share the same GO annotation, they originate a FN (*rounded squares*) by virtue of internal PPI (*solid lines*). In many cases (**B, E**)*,* it may happen that a protein has more annotations (*asterisk*) and is therefore assigned to more FN. (**C, F)** Eventually, two FN, which share crossing PPI (*dashed lines*) and/or proteins, are linked in a PG.

The algorithm initially defines as many FN as are the terms (in the ‘biological process’ vocabulary of GO) that are shared by at least two interacting proteins within a PPI network (step 2 of the algorithm pseudocode). This way, several FN are generated, each with a distinct protein content (Figure 1A). Frequently, however, a protein is annotated with more terms and is therefore assigned to more FN (Figure 1B). For instance, among the FN of the yeast peroxisome, the nodes that represent membrane assembly (node 45046) and the docking step of matrix assembly (node 16560) have distinct protein contents (Figure 1D), while the nodes that represent membrane assembly (node 45046) and inheritance (node 45033) have a partially overlapping content, i.e., the protein Pex3p (Figure 1E).

On one side, the multiplicity of annotations per protein may reflect the biological reality, in which the correspondence between functions and structures is commonly not a one-to-one relation. Rather, the same function can be supported by more structures and, conversely, the same structure can be devoted to more functions. Even at the molecular level, the same molecular structure (e.g., a protein) may participate in different functions, either because one copy of that protein encompasses functionally distinct domains or because more copies of that protein serve distinct functions (for instance, in different sub-cellular components). On the other side, most experimental procedures do not reliably guarantee that a function is affected by the annotated protein in a direct way (and not in an indirect way, i.e., by means of other functions, in a domino-like chain of reactions). In addition, in the hierarchical structure of GO, a protein is annotated not only with the specific term that defines a given function, but also with the more general parent terms of that function.

### Criteria for non-redundant protein-to-function assignment

**Figure 2:**
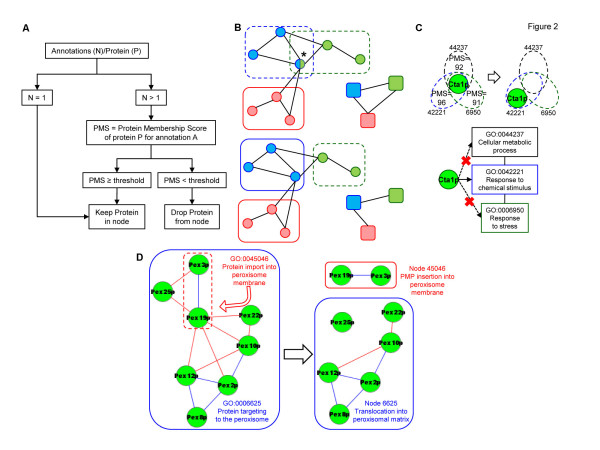
**Controlling annotation redundancy based on protein topology.** (***A***) Schematic overview of the procedure adopted for retaining a given protein only in those FN that satisfactorily overlap (as assessed by the PMS) the topological structures to which the protein belongs. In the example shown in (**B**), the protein doubly annotated with the *blue* and *green* terms is initially included into the two relevant FN (*rounded rectangles with dashed lines*). Subsequently, however, the protein is retained in the *blue* node (but excluded from the *green* node), because (compared with the *green* node) the *blue* node overlaps better the 3-protein clique (i.e., the triangle), to which the protein belongs. As a result, in the final PG, no edge is established between the *green* and *red* nodes. In (**C**), the example of the Cta1p catalase is shown, while (**D**) shows the procedure of enucleating a function from a FN, which then undergoes relabeling. See also Additional file 3 for the distribution of FN and edges at different NTS.

The algorithm takes several actions to decide whether the multiple annotations of a given protein do reflect its real participation in the annotating functions. The issue is critical, because redundant protein-to-function assignments would undermine our major aim to comply with the markov property, thus ensuring that the final PG is a coherent and structured representation of functions. A fairly obvious action is to select the most specific annotation out of a set of hierarchically ordered GO terms (step 2 of the algorithm pseudocode). The most important action, however, is to select only plausible inclusions of a protein into the FN, based on the topology of the PPI network (step 3 of the algorithm pseudocode). The rationale is that biological functions are based on the topological organization of their molecular components into modules, i.e., groups of molecules devoted to the same function, which are more densely connected among themselves than with the rest of the network [12-14]. Thus, the algorithm exploits a *protein membership score* (PMS), ranging from 0% to 100%, to measure the plausibility that a protein is member of the protein content of a FN (step 4 of the algorithm pseudocode). Specifically, the PMS reflects the ability of a FN to discriminate among distinct topological patterns of the PPI network, such as *k*-cliques (i.e., fully inter-connected sub-graphs of *k* proteins) and communities of *k*-cliques (i.e., unions of adjacent *k*-cliques), as defined in [10]. In practice, a protein is excluded from a FN, when another FN better overlaps the topological patterns to which the protein belongs (Figure 2A, B), unless its PMS is higher than a previously specified satisfactory threshold (here set to 95%). For instance, the peroxisomal catalase Cta1p is annotated with FN that refer to cellular metabolic processes (node 44237), responses to stress (node 6950) and responses to chemical stimuli (node 42221). However, Cta1p is excluded from nodes 44237 and 6950, but retained in node 42221, which likely represents the function most directly associated with Cta1p (Figure 2C). It should be noted that the procedure reduces, but does not exclude, FN with partly overlapping protein contents.

After these operations, it may happen that more FN have identical protein contents. In this case, the nodes are merged into one FN, and only the most specific term (i.e., the one with the greatest distance from the GO root) is retained as label. Otherwise, if the terms have the same specificity, they are all retained as label, with the resulting FN representing the union of the merged functions (step 5 of the algorithm pseudocode). For instance, the Tdh1p-2p-3p isozymes, which originate three FN with identical protein contents, are merged into one FN that retains the two most specific labels (glycolysis and gluconeogenesis; node 6094 + 6096), while the most generic term (glucose metabolic process; GO:0006006) is excluded.

It is also possible that a protein subset within a FN matches the protein content of another FN. In this case, the function associated with that protein subset is enucleated from the former FN, which should be viewed as excluding the enucleated function (step 5 of the algorithm pseudocode). For instance, a FN is annotated initially with term GO:0006625, which refers to protein targeting to the peroxisomes (Figure 2D). However, a subset of its proteins (Pex3p and Pex19p) matches the content of the FN annotated with term GO:0045046, which refers to the peroxisomal membrane assembly. Thus, the function of membrane assembly is enucleated from node 6625 and retained in node 45046.

In the end, not all the FN have a highly connected protein content. To focus on functions that correspond to the best defined structures of interacting proteins, each FN is given a *node topological score* (NTS), based on its ability to overlap a *k*-clique of proteins or a community of *k*-cliques (step 7 of the algorithm pseudocode). The NTS can be exploited to find an optimal threshold, below which a non linear marginal increment occurs in the number of edges or FN in the PG (see below, Additional file 3).

### Adapting the label of the function nodes to their biological meaning

The GO terms provide each FN with an initial label. Eventually, however, the label of each FN must be adapted to the functional role of its actual protein content and to its relations with other FN, to ensure specificity of definition (while preserving consistency with the original label). In general, GO terms must be adapted to the protein content of each FN, not only because the FN may undergo several procedures that modify its own original protein content, but also because the PPI network (which is restricted to a predefined biological domain) provides just a partial coverage of the whole interactome of the organism under study (such that the protein content of each FN corresponds only partially to the protein content of the relevant GO term). As an example of the impact of the applied procedure, after enucleation of Pex3p and Pex19p, the residual protein content of node 6625 refers more specifically to the translocation of cytosolic enzymes into the peroxisomal matrix. Thus, the original label of node 6625 ‘Protein targeting to peroxisome’ (corresponding to GO:0006625) was changed into the new label ‘Translocation into the peroxisome matrix’ (Figure 2D).

### From the function nodes to the process graph

On one hand, FN derive from annotations representing random variables that refer implicitly to an exhaustive set of alternative states, like ‘present’/‘absent’, ‘active’/‘inactive’ or a richer set of values. Therefore, a relation between two FN refers to a possible co-variation of their states. On the other hand, PPI link proteins that belong to either the same FN (‘internal PPI’) or different FN (‘crossing PPI’). While the former were used for defining the FN, the latter provide information about the mutual relations of the FN. Thus, an edge is initially established between two FN, if they are linked by a crossing PPI, provided that it was detected by a binary assay (Figure 1 C, F).

However, while the occurrence of a PPI provides evidence of a biochemical reaction, per se it is not deemed sufficient to infer a relation at a functional level. Actually, to focus on the biochemical reactions that more specifically support the hypothetical link between any two functions, functional links based on only one PPI are discarded (step 6 of the algorithm pseudocode). Furthermore, an edge is established between two FN, if they have a partially overlapping protein content. However, a single shared protein is not deemed sufficient to infer a relation between functions, mostly because more copies of the same protein might independently support the functions (step 6 of the algorithm pseudocode). For instance, no link is established between the FN representing peroxisome fission (node 16559) and fatty acid oxidation (node 19395), because the two FN share only the Pex11p protein (not shown).

Hereafter, we provide a description of three PG representations of well known cellular domains to systematically assess their validity. Specifically, each PG is revised to unveil both false and lacking relationships among any two FN, as well as to emphasize the compliance of larger portions of the graph with the markov property. For a detailed information and biochemical explanations, the reader is referred to the Additional file 4 and Additional file 5.

### The process graph of the *S. cerevisiae* peroxisome

**Figure 3:**
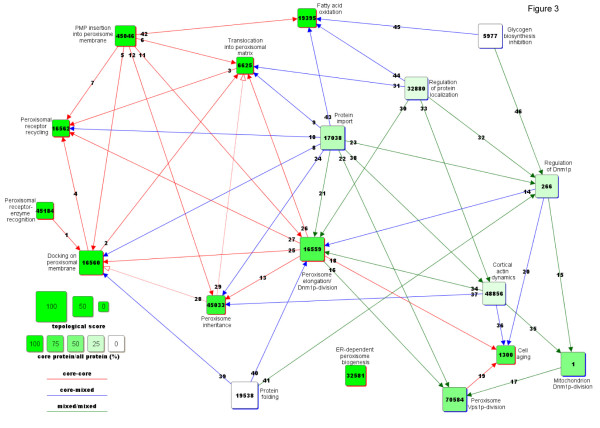
**The peroxisome process graph at high topological score.** The PG shows the FN that represent peroxisome-specific functions and extra-peroxisomal functions. Specifically, core and neighbor FN were chosen because of their highly connected protein content, as reflected in NTS ≥ 30 (core) or NTS ≥ 60 (neighbors). See also Additional file 6 and Additional file 4, for a detailed analysis of the FN and the edges, respectively.

The PG of the yeast peroxisome comprises 249 FN and 5,703 edges. Among the FN, 11 contain exclusively core proteins, 185 exclusively neighbor proteins, while 53 contain both core and neighbor proteins. For ease of analysis, FN have been selected based on NTS (Additional file 3) and protein type (i.e., core versus neighbor). Specifically, to focus on peroxisome-specific functions, FN were selected with NTS ≥ 30 and a core protein content of at least two thirds. Furthermore, to focus on other functions that may establish relations with the peroxisomal functions, FN were selected with NTS ≥ 60 and a core protein content of no more than one third. The resulting PG (Figure 3) consists of 18 FN (10 core and 8 peripheral; Additional file 6) and 46 edges (18 core-core, 14 core-neighbor and 14 neighbor-neighbor; Additional file 4).

### A process graph-based overview of peroxisome function

A brief description of the peroxisome PG shown in Figure 3 is provided here, while a detailed analysis can be found in the Additional file 4. First, it is known that the metabolic activity of the peroxisomal enzymes must be localized to the peroxisome matrix. Accordingly, the PG portrays the conditions that enable these activities and, in particular, the import of the enzymes from the cytosol (where they are synthesized) into the peroxisome matrix, i.e., the process of matrix assembly. Specifically, the enzymes are first recognized and bound by receptors in the cytosol (node 45184), so that the receptor-enzyme complexes can then dock onto the peroxisomal membrane (node 16560). In turn, docking allows the translocation of the enzymes into the matrix, across the peroxisomal membrane (node 6625). Once the enzymes are imported, the receptor is recycled back to the cytosol (node 16562) for another round of import. The graph also indicates that matrix assembly depends on membrane assembly, i.e., the insertion of Peroxisomal Membrane Proteins (PMP) into the peroxisome membrane (node 45046). Actually, once inserted, the PMP assemble to form the docking (node 16560), translocation (node 6625) and receptor recycling (node 16562) complexes.

Second, membrane assembly (node 45046) is also required for inserting PMP that mediate peroxisome fission (node 16559) and inheritance (node 45033). Fission (i.e., the formation of peroxisomes from pre-existing ones) refers to the elongation and subsequent division of the organelle, which requires the dynamin Dnm1p (node 16559). The same division factor is responsible for mitochondrion fission (node 1) and is similarly controlled in both peroxisomes and mitochondria (node 266). An unrelated system, which requires the dynamin Vps1p, controls selectively fission in peroxisomes (node 70584). Both division machineries (nodes 16559 and 70584) may influence cell aging (node 1300). As most of the fission-related factors must be imported into the peroxisomes, nodes 16559, 70584 and 266 depend on protein import (node 17038). The PG also portrays the dependence of inheritance (node 45033) on fission (node 16559). Actually, inheritance is the function whereby peroxisomes, which have been duplicated by fission, are delivered from the mother to the bud cell.

Third, the graph also captures regulatory functions, in particular of protein localization and stability. Thus, localization signals (node 32880) regulate peroxisome fission, by targeting to the peroxisomes regulators of elongation (node 16559), of Dnm1p (node 266) and of cortical actin (node 48856). Also, stability regulation involves the proteasome (node 19538), with possible effects on peroxisome fission (nodes 16559 and 266) and matrix assembly (node 16560). Finally, the PG highlights links between peroxisomes and metabolic functions, including fatty acid oxidation (node 19395), which depends again on membrane assembly (node 45046). In addition, other links, which involve Dnm1p regulation (node 266), suggest coordinated regulation of peroxisome fission (node 16559) and glycogen biosynthesis (node 5977), possibly in response to glucose availability.

### Presence of dubious edges and absence of expected edges in the peroxisome process graph

Some edges portray plausible (albeit not characterized) dependencies among functions, which call for experimental validation (as discussed in the next section). Few other edges, however, remain of dubious interpretation, as it may occur when a protein, which participates in different functions, is linked to proteins that participate in an additional function. For instance, Pex3p, which participates in membrane assembly (node 45046) and inheritance (node 45033), is linked to proteins that participate in docking (node 16560). As docking requires membrane assembly (and not inheritance), only the edge between nodes 45046 and 16560 (and not the edge between nodes 45033 and 16560) seems plausible (Figure 1E).

In contrast, some dependencies (albeit expected) are not portrayed by the edges of the PG, as it may occur when information is incomplete about protein interactions and/or annotations. For instance, concerning the interactions, even though it is established that peroxisomes can be formed from the ER (as represented in the PG by node 32581), the PPI underlying the ER-to-peroxisome connection are incompletely characterized. As a consequence, no edges in the PG link directly the peroxisomal nodes with node 32581. Furthermore, concerning the annotations, defective annotation of Pex5p with the term GO:0016562 results in the absence from the PG of an expected edge linking receptor recycling (node 16562) with receptor-dependent enzyme recognition (node 45184), as discussed in Additional file 4.

### Formulating experimentally testable hypotheses

Suggesting the direction of an edge between any two FN in a PG implies hypothesizing a causal dependence between the two represented functions. For instance, if node A points to node B, then function B depends on function A. Given that the primary source of evidence, i.e., the PPI network, offers several clues on the occurrence, but not the direction of causality, the algorithm elaborates an undirected graph that still requires additional biological knowledge to be fully specified as a directed graph. When specification of direction would yield directed cycles, standard techniques can be applied to obtain a DAG, leading to elimination of recursive relations, by redefining nodes as temporally ordered sequences of variables [15].

Converting an undirected graph into a DAG requires not only attributing directionality to the undirected edges but also removing those undirected edges that portray dependencies among two or more causal explanations, as long as they are deemed to be merely induced by the observation of common effects [8]. Edges, whose direction remains undetermined, originate multiple hypothetical markov equivalent DAG, each representing an experimentally testable conjecture. Whether a larger or smaller part of the DAG should be exploited to represent the experimental design, is a matter of convenience, as it is not always easy to assess the functional state of some nodes [7,16].

**Figure 4:**
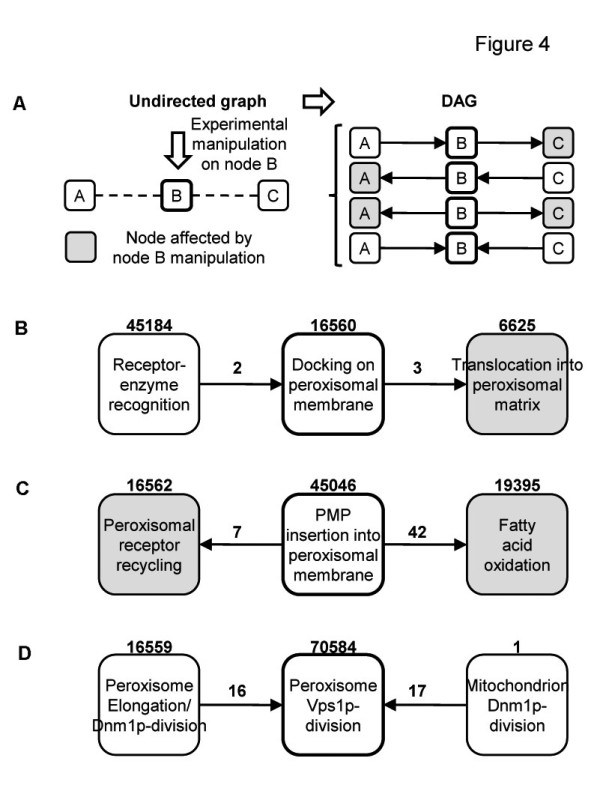
**Examples of experimentally-testable hypotheses.** (**A**) The general strategy and (**B-D**) different examples of experimentally-testable hypotheses derived from the PG of Figure 3 are shown. See also Additional file 7 for a list of selected DAG.

Here, we focus on undirected sub-graphs consisting of three nodes and two edges, which originate four hypothetical and testable DAG (Figure 4A). The experimental strategy requires manipulating one of the FN (node B) and assessing the state of the other two FN (A and C). Provided that specific manipulation and assessment are both feasible, the result allows selecting one of the possible DAG (Additional file 7). The following examples from the peroxisome PG indicate how our approach can be used to plan novel experiments (or to evaluate our graphical representation in the light of available data). First, to confirm the established sequence of events in peroxisome matrix assembly (Figure 4B), one might devise an experiment consisting of the manipulation (e.g., with blocking reagents) of docking (node 16560), which is expected to affect translocation (node 6625), while leaving enzyme recognition (node 45184) unaffected. Second, other experiments might be conceived to test the likely dependence of peroxisomal receptor recycling and fatty acid oxidation on membrane assembly (Figure 4C). In this case, available data might be used to corroborate the experimental design. Actually, manipulation of membrane assembly (node 45046), for instance by null mutation of *pex19*, primarily results in cytosolic mislocalization of several PMP, including Pex15p [17], which mediates receptor recycling (node 16562). Similarly, null mutation of *pex3* (node 45046) affects fatty acid oxidation (node 19395), possibly by altering localization of the PMP Pex11p [18]. Conversely, manipulation of nodes 16562 and 19395 (by null mutation of *pex15* and *pex11*, respectively) leaves PMP localization unaffected [17,19], thus strengthening the likely dependence of nodes 16562 and 19395 on node 45046. Third, other experiments might be devised to test the hypothetical dependence of the Vps1p-mediated fission of peroxisomes on the Dnm1p-mediated fission of both peroxisomes and mitochondria (Figure 4D). In partial support of this hypothesis, known genetic interactions (in particular, phenotype suppression) already suggest dependences among the fission-related FN. Specifically, manipulation of Vps1p-dependent fission (node 70584), by means of Vps1p over-expression, does not restore the fission defects (nodes 16559 and 1) of *dnm1* mutants, whereas manipulation of nodes 16559 and 1, by means of Dnm1p over-expression, does restore the fission defect of peroxisomes in *vps1* mutants [20].

### Process graph-based overview of the cellular bud

**Figure 5:**
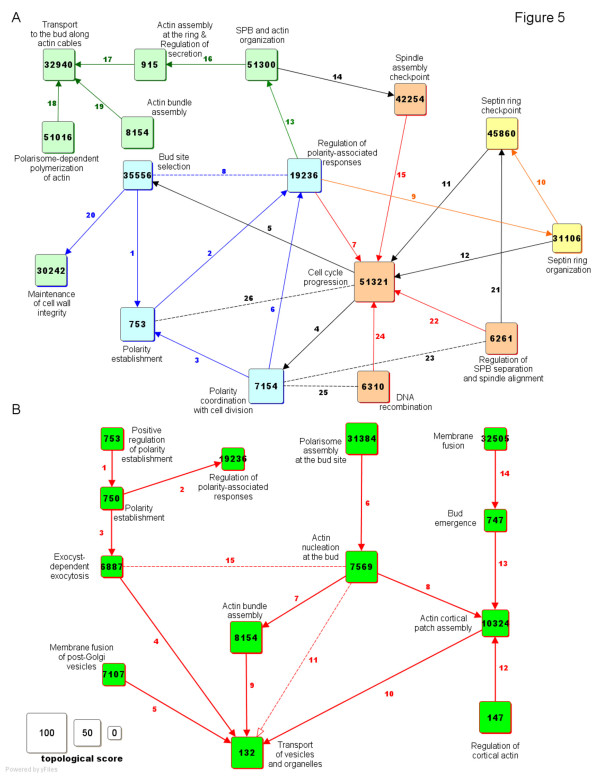
**The budding-related process graphs.** The PG shows the FN that represent functions related to the cellular bud (**A**) and to cell budding (**B**), two examples of a cellular component and a biological process in the budding yeast, respectively. See also Additional file 5 for a detailed analysis of the FN and the edges.

Lastly, in addition to the peroxisome, we have applied our method to other examples of PPI networks in the budding yeast. The proteins in these networks either localize to the cellular bud (Figure 5A) or participate in the process of cell budding (Figure 5B).

From a PPI network composed of 526 PPI and 154 proteins with the annotation ‘Cellular bud’ (GO:0005933 and child terms in the cellular component vocabulary of GO), a PG of 102 FN and 682 edges is generated. Setting a threshold NTS ≥ 55 produces a PG of 16 FN and 26 edges (Figure 5A), which is described briefly here (and in detail in Additional file 5). The PG represents functions that take place at the cellular bud in association with the polarization of the mother cell (*light blue*). Specifically, the Cdc42p-mediated establishment of polarity (node 753), which depends on the selection of the bud site (node 35556), activates in turn a kinase-based centre of regulation for polarity-related responses (node 19236), such as cytoskeleton remodelling along the mother-bud axis (*green*), ring formation at the bud neck (*yellow*) and cell division (*orange*). First, polarity induces both spindle reorientation and actin organization (node 51300), so that actin may assemble into filaments (node 915), which in turn favours polarized transport to the bud (node 32940). Transport also depends on the polarisome (node 51016) and on actin bundling (node 8154) for the correct orientation and strengthening of the filaments, respectively. Second, polarity, via the phosphorylation of septins, induces the assembly of a septin-based contractile ring around the neck of the bud (node 31106). Third, the PG shows that polarity is coordinated with the cell cycle in several ways. On one side, in G1 phase, polarity depends on the cell cycle, because Cdc28p (node 51321) inactivates the Cdc42p inhibitor Rga2p (node 7154). On the other side, in late G2/M phase, the cell cycle depends on polarity to allow entry in mitosis, because the Cdc42p effector Cla4p (node 19236) induces phosphorylation-mediated degradation of the Cdc28p inhibitor Swe1p (node 51321). The PG also shows that the cell cycle is regulated by polarity-related checkpoints for septin ring organization (node 45860), spindle assembly (node 42254) and spindle alignment (node 6261). Other accessory functions, such as DNA replication (node 6310) and cell wall remodelling (node 30242) are also shown.

### Process graph-based overview of cell budding

From a PPI network composed of 185 PPI and 72 proteins with the annotation ‘Cell budding’ (GO:0007114 and child terms in the biological process vocabulary of GO), a PG of 42 FN and 62 edges is generated. Setting a threshold NTS ≥ 27 produces a PG of 20 FN and 20 edges, which is fragmented into three clusters. The domain represented by the largest cluster (13 FN and 15 edges) is shown in Figure 5B and described briefly here (see also Additional file 5). The PG represents one of the key events of cell budding (i.e., polarized transport of vesicles and organelles from the mother cell to the bud) as a FN (node 132), together with its dependence on other FN, which are related to the underlying mechanisms of transport. First, polarized transport of vesicles (node 132) depends on the fusion of post-Golgi exocytic vesicles with the plasma membrane (node 7107), as well as on the establishment of a specific site of fusion at the plasma membrane, where the exocyst complex localizes (node 6887). Exocyst localization depends on the Cdc42p-mediated establishment of polarity (node 750), which depends on upstream regulators (node 753). In turn, polarity establishment (node 750) regulates other budding-related responses, such as the assembly of a septin-based ring around the bud neck (node 19236). Second, polarized transport of organelles requires the formation of polymeric actin cables along the mother-bud axis and their anchoring at a cortical actin patch in the bud. Specifically, nucleation of actin monomers (node 7569) depends on the assembly of the polarisome complex at the site of bud emergence (node 31384). Then, actin polymerization induces the assembly of both actin bundles (node 8154) and a patch of cortical actin (node 10324). The patch also depends on additional upstream regulators (node 147), as well as on the growth of the bud (node 747), which in turn depends on membrane fusion events (node 32505). Finally, these actin structures enable the polarized transport (node 132).

Given that polarity establishment is known to induce actin polarization [21], an edge from node 19236 to node 32940 (in the cellular bud PG of Figure 5A), as well as an edge from node 750 to node 7569 (in the cell budding PG of Figure 5B) was expected. The edge, however, cannot be but missing, since polarity-dependent regulators of actin, like Arp2p (or other proteins of the Arp2/3 complex), were not annotated with the polarity-related GO terms used to select the proteins of the PPI networks, despite the fact that Arp2p is an effector of the polarity regulator Las17p [22,23]. This observation might suggest the usefulness of extending the PPI selection to the first degree neighbours of the core proteins of the domain of interest, as likely means to reducing missing annotations, even though the extension likely increases the density of the edges in the PG (see Additional file 8).

## Discussion

This method combines information on the interactions and functions of the proteins that belong to a domain of biological interest (e.g., a cellular organelle or a biological process), with the goal of converting a functionally annotated PPI network into a PG, i.e., a compact and coherently structured representation of dependencies among biological functions. The goal is challenging, as available information about the protein-to-function relations does not guarantee that a protein under examination does indeed participate directly in the annotated function. As edges between functions are based on the PPI among the proteins that these functions annotate, it follows that redundant protein-to-function assignment inevitably produces redundant edges among the corresponding FN. Thus, throughout the study, it has been our main concern to ensure that a direct edge between two FN could be established, only if intermediate functions were unlikely to occur. Otherwise, the resulting PG would be a mere assembly of coupled functions and not a coherent and compact representation of the way functions cooperate in supporting complex biological activities. In addition, a redundant PG would be of limited usefulness for planning the smallest set of experimental interventions that can be made on a function, when one desires to impact on target functions. To achieve compact representations, we took the following considerations into account. First, FN are expected to map onto a PPI network the correspondence between proteins and annotations. Second, such mapping is expected to represent the most extensive coverage of the PPI network with the least degree of overlap between FN, provided that one can exclude the annotations of those proteins that support only indirectly the annotated functions. Third, molecules more typically contribute to biological functions as highly inter-connected (or ‘modular’) assemblies, rather than as unconnected elements [12]. Within PPI networks, for instance, functional and topological modules display significant overlap [10,24]. Thus, based on these considerations, the algorithm we have devised introduces a topologically-driven prioritization that selects only plausible inclusions of a protein into a FN, as quantified by its PMS, i.e., a score that reflects the ability of a FN to discriminate among the topological patterns of the PPI network, to which the protein belongs.

The method has been applied to two cellular components (i.e., the peroxisome and the cell bud) and one biological process (i.e., cell budding) in *S. cerevisiae*, which are well characterized domains and thus suitable for validation purposes. On one hand, well characterized causal dependencies among functions (e.g., dependence of peroxisome matrix on membrane assembly) have confirmed that the method specifically highlights important relations. On the other hand, less obvious dependencies (e.g., those among different fission-related mechanisms in peroxisomes and mitochondria) have revealed the heuristic power of this method and its usefulness in formulating testable hypotheses. It should be noted that the peroxisome-centered PPI network has been extended to the first neighbors of the peroxisome core proteins, because we wanted to highlight the wider biological landscape that ideally surrounds the organelle. The inclusion of non-peroxisomal proteins is justified by the observation that almost half of the core proteins are annotated (in the cellular compartment vocabulary of GO) with terms related not only to the peroxisome but also to mitochondrion, ER and nucleus (Additional file 1), the organelles that interact functionally with the peroxisome [25,26]. Clearly, some multiple annotations of the same protein simply refer to the existence of sub-cellular distinct (and functionally unrelated) pools, such as the peroxisomal and nuclear pools of the dynamin Dyn2p. Nevertheless, other multiple annotations suggest that a protein may change its sub-cellular location, at least under specific conditions. For instance, Pex11p relocates from the ER to the peroxisome, when peroxisomes are induce to proliferate in response to oleate [27].

Numerous studies have analyzed the relation between molecules and functions. In particular, one of the major aims of many bioinformatics studies has been to infer the function of uncharacterized genes based on comparisons with characterized genes, such as sequence similarity [28], co-occurrence in genomic clusters [29], co-evolution in different species [30] and co-expression patterns [31]. Also the PPI have been used to infer the function of uncharacterized proteins, based on the most frequent annotations of their protein interactors [4,32-34]. Like these ‘guilt by association’ methods, also our approach builds on the assumption that proteins often interact mutually to contribute to the same function. Furthermore, all these studies (including ours) deploy a non-directional annotated network as input, sometimes designed ‘functional linkage network’ [35], in which nodes correspond to molecules, while edges correspond to different types of functional connection between molecules [36].

Few studies, however, have moved beyond the immediate aim of inferring protein-function binary associations to the ultimate aim of inferring structured dependencies among functions, which can be displayed in a markov graph of connected functions. An earlier study established a link between a given pair of functions, anytime a PPI had been detected between the proteins annotated with the two functions [4]. A more recent study has elaborated on this method, by selecting statistically enriched pairs of functions, as defined by the probability that two sets of proteins (annotated with two distinct functions) establish more PPI between themselves than it can be expected by chance [5]. Our method differs considerably from these earlier studies, as it retains any annotation that is shared by two interacting proteins within the PPI network, leaving to the topological analysis the task to define the FN, whose relationships may satisfy the markov property. This way, the selected and prioritized protein assignments to the FN are expected to refer truly to the functions that are directly impacted by the protein. As an example of how our approach differs from the previous studies, consider a 3-node sub-graph composed of FN related to the GO functional annotations of protein import (GO:0017038), PMP insertion into the peroxisome membrane (GO:0045046) and peroxisome organization (GO:0007031). While a previous study linked the three nodes with three edges [5], our algorithm establishes only two edges (one between nodes 17038 and 45046 and another between nodes 45046 and 7031), but not the edge between nodes 17038 and 7031, because it would violate the markov assumption, given that protein import (node 17038) contributes to peroxisome organization (node 7031) only indirectly, i.e., by enabling PMP insertion (node 45046). Furthermore, our study also contrasts with an important implication of the earlier study, which advocated re-engineering the GO database by complementing the GO hierarchy with the links inferred from the functional linkage graphs [5]. Alternatively, we propose to adapt the semantics of the GO annotations to the level of detail that characterizes the domain of interest, mostly based on the real protein content of the FN. A more detailed comparison of graphs that represent similar domains but are obtained with different methods can be found in Additional file 8.

Clearly, our method can be applied to different domains of biological interest in different model organisms, even though some words of caution should be added. First and foremost, inaccurate and/or defective datasets of protein interactions and functions will certainly affect the quality of the PG representation. In our experience with *S. cerevisiae*, even after revising carefully the PG, we found just a limited number of false positives and false negatives. Nevertheless, we cannot exclude that results might be less accurate, should the algorithm be applied to other organisms that are not so extensively and accurately characterized as the budding yeast. Second, other features of the starting PPI network should be taken into account, including the choice of a cell type-specific repertoire of proteins (in the case of multi-cellular organisms) and the size of the PPI (to ensure computational tractability). Third, it should be pointed out that labor-intensive analysis is required to verify the consistency of the PG with current biological knowledge and to define the causal directionality of its undirected edges. It should also be taken into account that just a minor fraction of the physical interactions that are reported in the PPI databases have an annotation of biochemical directionality (e.g., kinase-dependent phosphorylation of a substrate). For instance, out of 106,230 PPI reported as ‘physical interactions’ in the 3.1.83 release of BIOGRID, more than 94% of PPI (100,388 PPI) has no annotation. Only less than 6% of the remaining PPI has the annotation ‘phosphorylation’ or other types of modifications. Furthermore, in many of these cases, the annotated modification has been detected in a biochemical assay without functional characterization. Finally, many PPI refer to physical interactions that are non-directional in nature (e.g., interactions among structural proteins).

In conclusion, with all the caveats related to incomplete knowledge, the herein reported data suggest that, even when the PPI structures that underlie a function are only partially known, it is nevertheless possible to regard functions as black boxes with only known inputs and outputs, to obtain non-redundant graphical representations of complex biological systems. In addition, our efforts indicate that the graph we obtain can be helpful in carefully designing experimental studies, provided that specific manipulation and measurement of the portrayed functions are feasible.

## Conclusions

The major problem with the idea of converting PPI networks (of interacting and functionally annotated proteins) into PG (of inter-dependent FN) is that several proteins have multiple annotations. Faced with this challenge, we reasoned that the final PG could be non-redundant, if only the proteins that participate directly in a given function are included in the related FN. Furthermore, we surmised that topological features (e.g., the presence of highly inter-connected protein clusters within the starting PPI network) might help approaching structured and non-redundant understanding of molecular function. Thus, an algorithm was developed that prioritizes inclusion of proteins into the FN that best overlap protein clusters. Specifically, the algorithm identifies FN (and their mutual relations), based on the topological analysis of the starting PPI network. Applying the algorithm to different domains of biological interest (i.e., the *S. cerevisiae* peroxisome, cellular bud and cell budding) has shown that the method is suitable for formulating testable and mechanistic hypotheses about the existence of dependencies among functions.

## Methods

### Assembly of the PPI network

The PPI network is assembled starting from the core proteins that characterize the domain of interest (e.g., the peroxisome, cellular bud and cell budding). Specifically, these proteins are the gene products (as verified open reading frames) that can be retrieved from the Saccharomyces Genome Database (SGD) [37], using the ‘Advanced Search’ option, with limit to the GO-Slim terms ‘Peroxisome’ (GO:0005777), ‘Cellular bud’ (GO:0005933) or ‘Cell budding’ (GO:0007114). Then, the list of gene products is used as query to retrieve from the SGD database the PPI (‘physical interactions’) that these proteins engage in, using the ‘Batch Download’ search. A similar search is then performed to retrieve the PPI that occur among the interactors of the core proteins.

### Algorithm pseudocode

A graph G = (**V, E**) is a pair made by a finite set V = {V_1_, V_2_, . . ., V _K_} of nodes and a collection of edges **E** ⊂ **V** × **V**. Sets are indicated either in bold or within braces, the number of their elements by indicating them within vertical lines. Available data consist of two graphs, namely, the PPI = (**prot**, **J**) and the DAG GO = (**annot**, **H**). The set **E** defines which nodes are linked by an edge, so that E_i,j_ means that node V_i_ is linked to V_j_. When a graph is a DAG, edges are oriented, so E_i,j_ ≠ E_i,j_. The subset **V**_**k**_ ⊂ **V** of nodes originating arrows reaching the node V_j_ is called parents set, *pa*(V_j_). A directed path is a path in which edges always meet head-to-tail. The ancestors set *anc*(V_i_) of node V_i_ contains nodes located on directed paths reaching V_i_. The set of annotations of a protein *i* as derived from GO is indicated by *annotations*(prot_i_). By reverse, the set of proteins annotated with annotation *k* is indicated by *proteins*(annot_k_). Other set of annotations are labelled as **annot**_**X**_, with X indicating a set of proteins. The protein content of a FN_i_ or a cluster of proteins C_z_ is labelled as **prot**_FNi_ and **prot**_Cz_, respectively. Complementary sets of proteins are indicated by the superscript *C*, so that **prot**_FNi_^*C*^ and **prot**_Cz_^*C*^ are the proteins in prot, but not in FN_i_ and C_z_, respectively.

#step 1: data loading

Get the PPI = (**prot**, **J**), the GO = (**annot**, **H**) and the set of clusters of proteins

C≡ {C_j_} : C_j_≡ **prot**_cj_ ⊆ **prot.**

#step 2: initial annotations of proteins

**for all** prot_v_ ∈ **prot**

**annot**_v_← Ø

**for all** annot_a_ ∈ *annotations*(prot_v_)

**for all** annot_b_ ∈ *annotations*(prot_v_), b>a

**if annot**_a_ ∉*anc* (annot_b_)

**then annot**_v_ ← **annot**_v_ ∪ annot_a_

end

end

end

#step 3: initial annotations’ mapping of PPI (creation of FNs)

PG= (**FN**, **E**), with **FN** ≡ Ø, **E**≡ Ø

**for all** prot_v_ ∈ **prot**

**for all** prot_w_ ∈ **prot**, w>v

**if** J_v,w_∈**J then do**

**if** (**annot**_v_ ∩ **annot**_w_) ∪ (*pa*(**annot**_v_) ∩ *pa*(**annot**_w_))≠Ø) **then do**

**annot**_z_ ← (**annot**_v_ ∩ **annot**_w_) ∪ (*pa*(**annot**_v_) ∩ *pa*(**annot**_w_))

**for all** annot_i_ ∈ **annot**_z_

**if** annot_i_ ∉ **FN then**

FN_i_←annot_i_; **prot**_FNi_←{prot_v_,prot_w_}; **FN**← **FN**∪FN_i_

Else

**prot**_FNi_←**prot**_FNi_∪{prot_v_,prot_w_};

end

end

end

end

end

# step 4: refinement of annotations mapping of PPI based on PPI topology

**for all** FN_i_∈ **FN**

**for all** prot_v_ ∈ **prot**_FNi_

PMS(prot_v_, FN_i_) ←0

**for all** C_z_ ∈ C : prot_v_ ∈ C_z_

(1) $$PMS=\max\left(PMS\;\text{,}\;\frac{\left|{\text{prot}}_{{\text{FN}}_{\text{i}}}\cap {\text{prot}}_{{\text{C}}_{\text{z}}}|+|{\text{prot}}_{{\text{FN}}_{\text{i}}^{C}}\cap {\text{prot}}_{{\text{C}}_{\text{z}}^{C}}\right|}{\left|{\text{prot}}_{{\text{FN}}_{\text{i}}}\cap {\text{prot}}_{{\text{C}}_{\text{z}}}|+|{\text{prot}}_{{\text{FN}}_{\text{i}}^{C}}\cap {\text{prot}}_{{\text{C}}_{\text{z}}}|+|{\text{prot}}_{{\text{FN}}_{\text{i}}^{C}}\cap {\text{prot}}_{{\text{C}}_{\text{z}}^{C}}|+|{\text{prot}}_{{\text{FN}}_{\text{i}}^{C}}\cap {\text{prot}}_{{\text{C}}_{\text{z}}^{C}}\right|}\right)\times 100$$

end

end

end

**for all** FN_i_∈ FN

PMS*←max(PMS(**prot**_FNi_))

for all **prot**_v_ ∈ **prot**_FNi_

**if** PMS(prot_v_, FN_i_)<PMS* & PMS< PMS_threshold_**then**

**prot**_FNi_← **prot**_FNi_/prot_v_

end

end

# step 5: elimination and redefinition of redundant FNs

**for all** FN_k_∈ **FN**

**for all** FN_h_∈ **FN**, h>k

**if prot**_FNk_≡**prot**_FNh_**then FN** ← **FN**/FN_k_

**if prot**_FNk_⊆**prot**_FNh_ then prot_FNh_ ← prot_FNh_/prot_FNk_

end

end

# step 6: edges among FNs

**for all** FN_k_∈ **FN**

**for all** FN_j_∈ **FN**, j>k

**if** |**prot**_FNk_ ∩ **prot**_FNj_| >1 **then E**← **E**∪E_k,j_

cnt←0

**for all** prot_v_ ∈ **prot**_FNk_

**for all** prot_w_ ∈ **prot**_FNj_

**if** J_v,w_∈ **J then** cnt ← cnt +1

end

end

**if** cnt >1 **then E**← **E**∪E_k,j_

end

end

# step 7: PG reduction

**for all** FN_k_∈ FN

NTS(FN_k_) ←0

**for all** C_z_ ∈ C

(2) $$NTS({FN}_{k})=\max\left(NTS({FN}_{k})\text{,}\;\frac{\left|{\text{prot}}_{{FN}_{k}}\cap {\text{prot}}_{C_{z}}\right|}{\left|{\text{prot}}_{{FN}_{k}}\cap {\text{prot}}_{C_{z}}|+|{\text{prot}}_{{FN}_{k}}\cap {\text{prot}}_{C_{z}^{C}}|+|{\text{prot}}_{{FN}_{k}^{C}}\cap {\text{prot}}_{C_{z}^{C}}\right|}\right)\times 100$$

end

**if** NTS(FN_k_)< NTS_threshold_**then FN** ← **FN**/FN_k_

end

### Inferring the direction of the edges within the PG: general criteria and examples

Inferring the direction of each edge in the undirected PG (produced by the algorithm) is a manual procedure, which is performed by an expert investigator in the light of biological knowledge. Given the heterogeneous nature of current biological knowledge, the procedure relies on different types of evidence (and corresponding operational criteria). Considering two hypothetical functions A and B, the direction of the edge linking A and B (and pointing from A to B) is inferred (and expressed in the format ‘B depends on A’) according to one of the following rules. First, direct experimental evidence indicates that manipulation of A affects B (rule 1) or that the main component(s) of A affect the main component(s) of B (rule 2). Second, current understanding of the specific domain (e.g., yeast peroxisome) indicates that B logically implies A (rule 3), or that event A precedes event B (rule 4) or that the main component(s) of A affect the main component(s) of B (rule 5). Third, current understanding of cell biology suggests that A might affect B (rule 6). Hereafter, some links from the peroxisome PG (Figure 3) are discussed to exemplify the six rules.

(*Rule 1*) Link 13 indicates that peroxisome inheritance (node 45033) depends on Dnm1p-dependent peroxisome fission (node 16559). The inference is based on direct experimental evidence that manipulation of A (defective fission, with presence of non-divided peroxisomes in mother cells lacking the fission factor Pex11p) affects B (defective inheritance, with absence of inherited peroxisomes in the bud).

(*Rule 2*) Link 30 indicates that Dnm1p-dependent peroxisome fission (node 16559) depends on the regulation of protein localization (node 32880). The inference is based on the experimental evidence that the main component of A (the Pho85p kinase) affects (phosphorylates) the main component of B (the fission factor Pex11p), which would otherwise fail to localize to the peroxisome.

(*Rule 3*) Link 5 indicates that the docking of receptor-cargo complexes on the peroxisomal membrane (node 16560) depends on the assembly of the peroxisomal membrane (node 45046). The inference is based on current understanding of the specific domain, according to which B (docking) logically implies A (assembly of docking proteins).

(*Rule 4*) Link 2 indicates that translocation of receptor-cargo complexes across the peroxisomal membrane (node 6625) depends on their docking onto the outer surface of the peroxisomal membrane. The inference is based on current understanding of the specific domain, according to which event A (docking) precedes event B (translocation).

(*Rule 5*) Link 31 indicates that translocation (node 6625) depends on the regulation of protein localization (node 32880). The inference is based on current understanding of the specific domain, according to which the main component of A (the Pho85p kinase) affects (phosphorylates) the main component of B (the translocation factor Pex10p).

(*Rule 6*) Link 18 indicates that cell aging (node 1300) depends on peroxisome fission (node 16559). The inference is based on current understanding of cell biology that oxidative metabolism (like the one occurring in peroxisomes) may affect aging.

### Labeling the function nodes

The initial labels of the FN (expressed as GO terms) can undergo manual relabeling, if one (or more) of the following instances occurs. First, a FN has been enucleated from another FN. Second, more FN with identical protein content have been merged into a single FN. Third, the proteins of the predefined domain provide just a minor coverage of the proteins annotated by the GO term. In these instances, a new label is added provided that it represents more appropriately the actual function of the protein content of the FN (and/or its relations with other FN in the PG). Furthermore, it should embed (at least implicitly) some reference to the definition of the original GO term.

## Abbreviations

DAG, Directed Acyclic Graph; FN, Function Node; NTS, Node Topological Score; PG, Process Graph; PMS, Protein Membership Score; GO, Gene Ontology; PMP, Peroxisomal Membrane Proteins; PPI, Protein-Protein Interaction; SGD, Saccharomyces Genome Database.

## Competing interests

The authors declare that they have no competing interests.

## Authors’ contributions

Both authors conceived the study, discussed the results and wrote the paper. DL developed the algorithm, GB carried out the PG analysis. All authors read and approved the final manuscript.

## Supplementary Material

Additional file 1 **The proteins of the peroxisomal PPI network (xls).** The table reports all the core and neighbor proteins of the peroxisomal PPI network shown in Additional file 2, with the annotation(s) in the ‘cellular component’ ontology of GO (see last page of the table for explanation). The annotations are ‘Yeast GO Slim’ terms, except the ones in *italics*, which are child terms of the slim term GO:0005777 (‘Peroxisome’). *k* is the connectivity degree of each protein, i.e., its number of direct neighbors in the network. Click here for file

Additional file 2 **The peroxisomal PPI network (jpg).** The PPI network comprises peroxisomal core proteins and their direct neighbors (*green* and *yellow circles*, respectively), as well as the PPI that have been detected by binary (*red lines*) or cluster (*blue lines*) assay (see also Table 1 of the main text). The *size* of each node is proportional to the *k* value of the corresponding protein. Click here for file

Additional file 3 **Node and edge distribution in the peroxisome PG at different NTS (jpg).**The number of edges (*top*) and FN (*bottom*) in the peroxisome PG is shown as a function of the NTS. Click here for file

Additional file 4 **FN and edges of the peroxisome PG (pdf).** The file describes the FN and edges of the peroxisome PG (displayed in Figure 3 of the main text), the physical links underlying the edges (crossing PPI and/or shared proteins), as well as their biochemical basis and biological significance [38-75]. Click here for file

Additional file 5 **FN and edges of the cellular bud and cell budding PG (pdf).** The file describes the FN and the edges of the cellular bud and cell budding PG (displayed in Figure 5A and Figure 5B of the main text, respectively), the physical links underlying the edges (crossing PPI and/or shared proteins), as well as their biochemical basis and biological significance [76-94]. Click here for file

Additional file 6 **The FN of the peroxisome PG (jpg).** The FN of the peroxisome PG (displayed in Figure 3 of the main text) are shown with their protein contents, definitive labels and NTS. As in the Additional file 2, *green* and *yellow circles* represent core and neighbor proteins, respectively. Also, *red* and *blue lines* represent PPI detected by binary or cluster assays, respectively. Click here for file

Additional file 7 **Directed acyclic graphs in the peroxisome PG (xls).** The table reports the node identity of the different types of DAG shown schematically in Figure 4A of the main text. Click here for file

Additional file 8 **Comparative analysis (pdf).** The file describes the network analysis-based comparison of PG obtained with our method and with the method described in reference 5. Click here for file
