# Supplementary figures and images for: From networks of protein interactions to networks of functional dependencies
Source: BMC Syst Biol. 2012 May 20;6:44. doi: 10.1186/1752-0509-6-44 (PMC3434018; doi:10.1186/1752-0509-6-44)

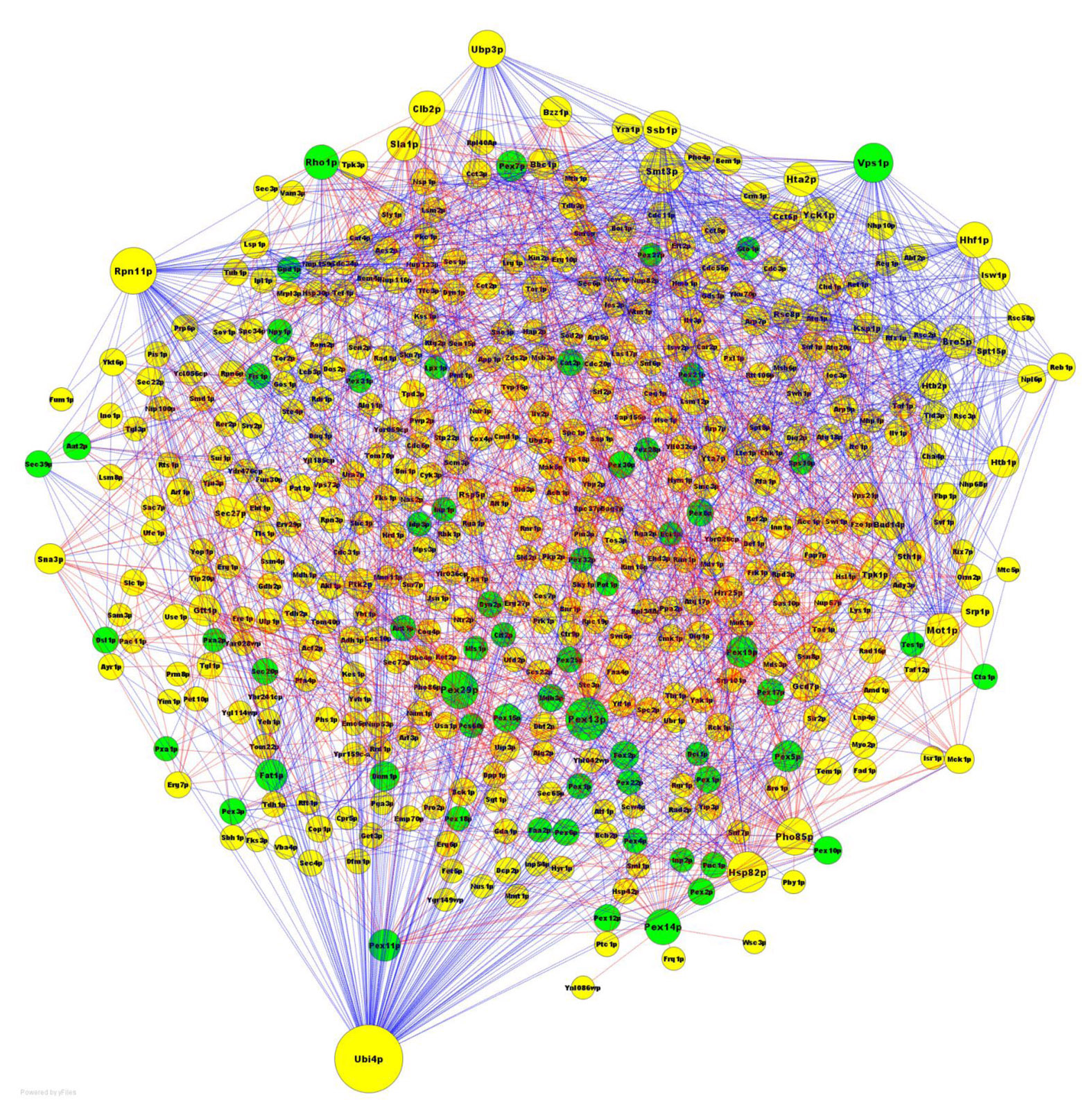

Supplement: Additional file 2 — The peroxisomal PPI network (jpg). The PPI network comprises peroxisomal core proteins and their direct neighbors (green and yellow circles, respectively), as well as the PPI that have been detected by binary (red lines) or cluster (blue lines) assay (see also Table 1 of the main text). The size of each node is proportional to the k value of the corresponding protein. [file 1752-0509-6-44-S2.jpeg]

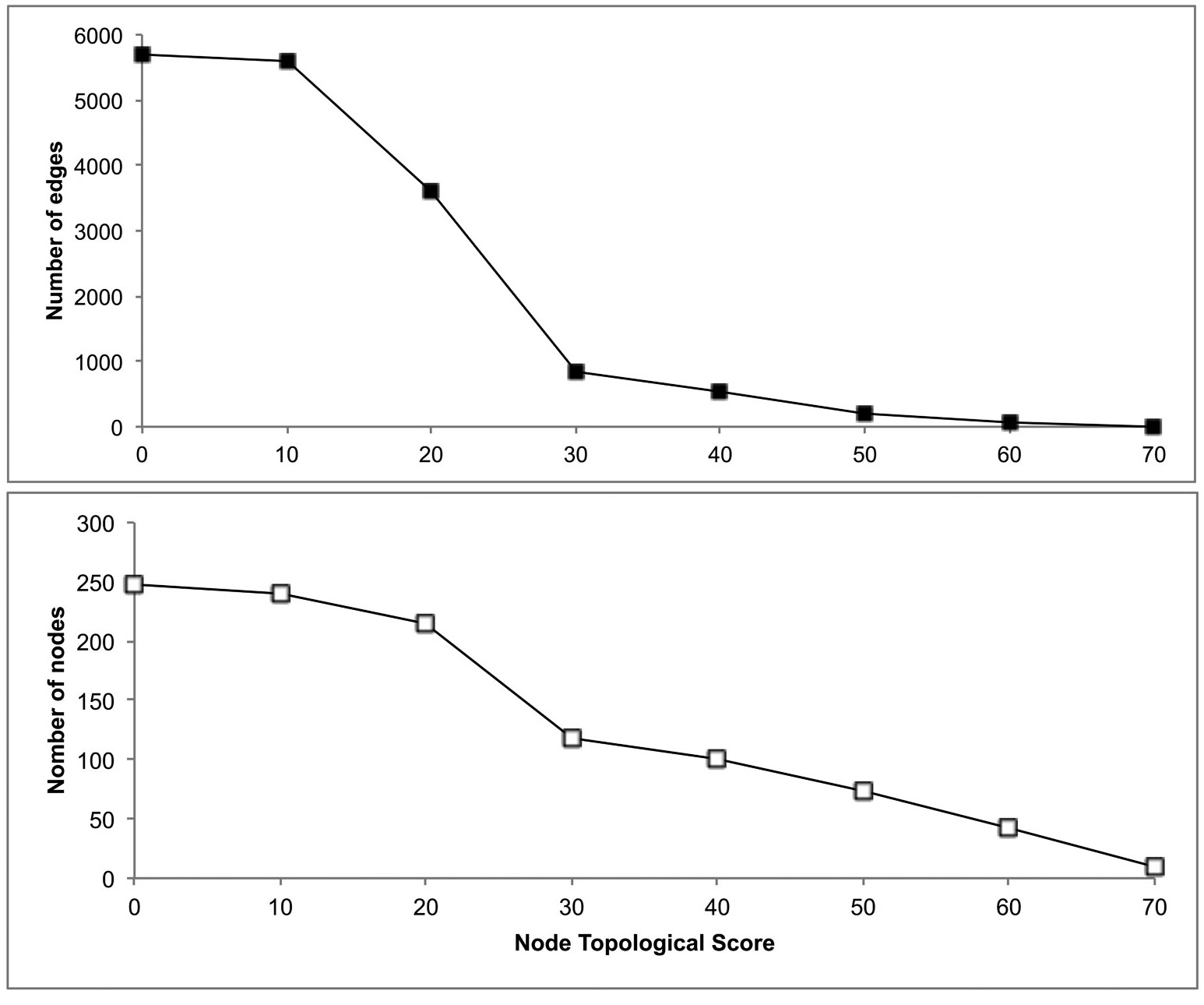

Supplement: Additional file 3 — Node and edge distribution in the peroxisome PG at different NTS (jpg).The number of edges (top) and FN (bottom) in the peroxisome PG is shown as a function of the NTS. [file 1752-0509-6-44-S3.jpeg]

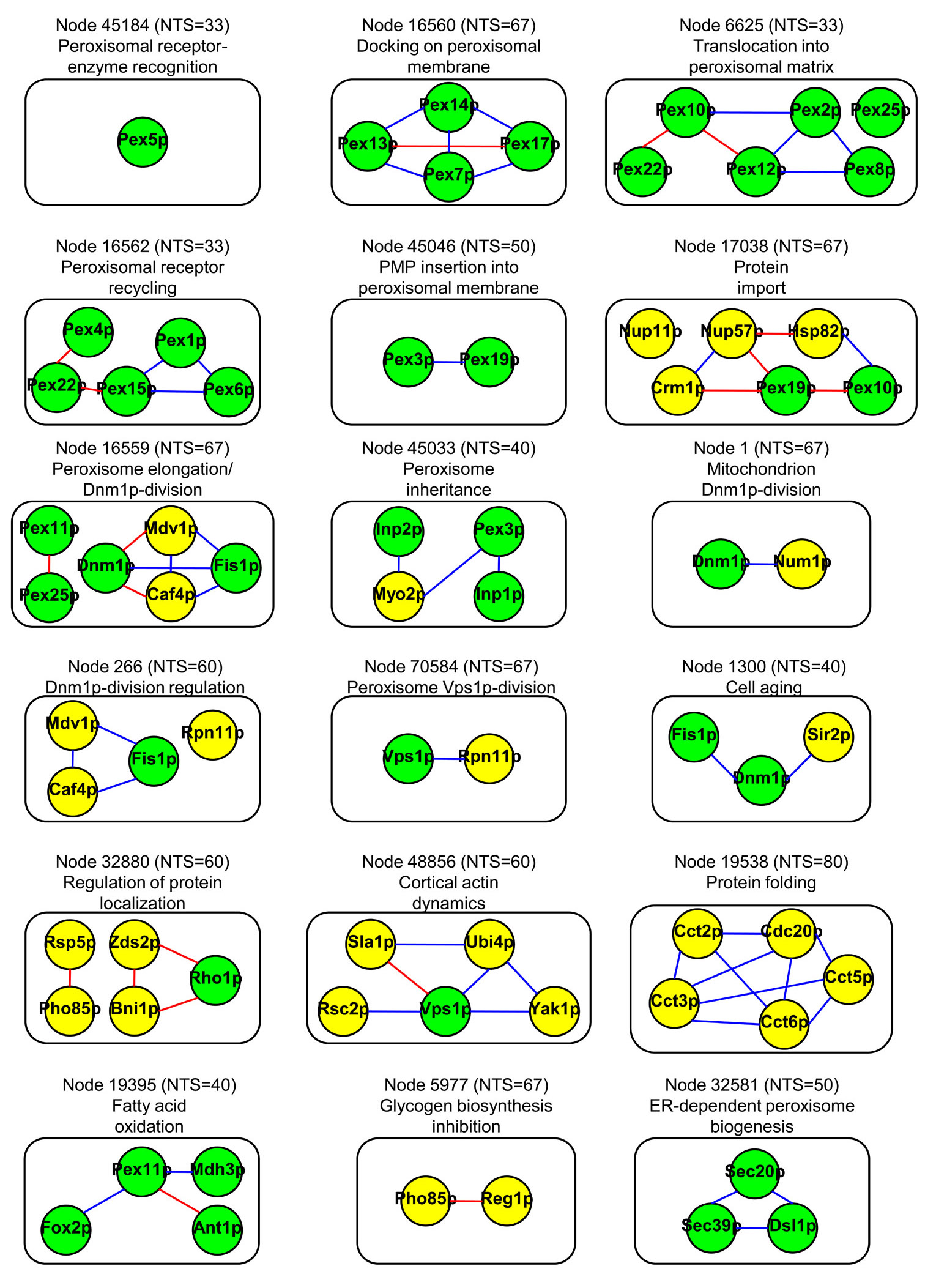

Supplement: Additional file 6 — The FN of the peroxisome PG (jpg). The FN of the peroxisome PG (displayed in Figure 3 of the main text) are shown with their protein contents, definitive labels and NTS. As in the Additional file 2, green and yellow circles represent core and neighbor proteins, respectively. Also, red and blue lines represent PPI detected by binary or cluster assays, respectively. [file 1752-0509-6-44-S6.jpeg]
