# Supplementary material for: From networks of protein interactions to networks of functional dependencies
Source: BMC Syst Biol. 2012 May 20;6:44. doi: 10.1186/1752-0509-6-44 (PMC3434018; doi:10.1186/1752-0509-6-44)
Supplement: Additional file 4 — FN and edges of the peroxisome PG (pdf). The file describes the FN and edges of the peroxisome PG (displayed in Figure 3 of the main text), the physical links underlying the edges (crossing PPI and/or shared proteins), as well as their biochemical basis and biological significance [38-75]. [file 1752-0509-6-44-S4.doc]

**The peroxisome PG**

Part I of this file describes in detail the peroxisome PG, which is shown in Figure 3 of the main text. Part II reports the original labels of the individual FN and the way they may have been relabeled. Finally, Part III reports the individual edges and the criteria whereby they may have been defined as directional in the PG.

**Part I: Description of the PG**

**1. Assembly of peroxisome matrix and membrane**

Peroxisomes are composed of a matrix surrounded by a membrane. To perform their metabolic functions, peroxisomes must recruit into the matrix various cytosolic enzymes. Four FN in the PG of Figure 3 annotate proteins that, as described below, participate in the multi-step process of matrix assembly (often as components of multi-protein complexes).

**Link 1 45184 > 16560 = Peroxisomal receptor-enzyme recognition > Docking on peroxisomal membrane**

Crossing PPI: Pex5p_Pex13p

Depending on the peroxisome targeting sequence of the enzyme, either the type 1 receptor Pex5p [38] or the type 2 receptor Pex7p [39] recognizes and binds the enzyme in the cytosol. Then, the receptor-enzyme complex docks onto the peroxisomal membrane. The type 1 receptor originates node 45184 ‘Peroxisomal receptor-enzyme recognition’, while the docking complex (which comprises the PMP Pex13p, Pex14p and Pex17p) originates node 16560 ‘Docking on peroxisomal membrane’. The Pex5p_ Pex13p PPI connects nodes 45184 and 16560 (**link 1**). Link 1 has been interpreted as directional (with node 16560 being dependent on node 45184), because interfering with enzyme recognition (node 45184) is expected to prevent docking (node 16560), whereas interfering with docking should not interfere with enzyme recognition.

Experimental support for this contention, however, is incomplete. On one side, deletion of either Pex5p [40] or Pex13p [41] does affect enzyme import into the matrix, i.e., the final outcome of matrix assembly. On the other side, mutating residue F208 (in the WXXQF sequence of Pex13p, i.e., the binding site for Pex5p) does not affect Pex5p localization to the peroxisome [42], even though the Pex5p_Pex14p PPI (which is not reported as crossing PPI, because detected by cluster assay) might compensate for the perturbation of the Pex5p_Pex13p PPI.

Furthermore, it should be noted that, in contrast to the type 1 receptor, type 2 receptor-dependent recognition and binding is less clearly identifiable in the PG, because the type 2 receptor Pex7p is annotated in node 16560, while its co-receptors Pex18p and Pex21p are included in node 16558, which is absent from the graph because of low NTS.

**Link 2 16560 > 6625 = Docking on peroxisomal membrane > Translocation into peroxisomal matrix**

Crossing PPI: Pex13p_Pex10p; Pex14p_Pex10p; Pex17p_Pex10p; Pex7p_Pex8p

**Link 3 6625 > 16562 = Translocation into peroxisomal matrix > Peroxisomal receptor recycling**

Crossing PPI: Pex10p_Pex4p; Pex10p_Pex1p; Pex10p_Pex6p; Pex10p_Pex15p; Shared Protein: Pex22p

Other proteins (i.e., the linker Pex8p and the members of the RING finger complex Pex2p, Pex10p and Pex12p), which originate node 6625 ‘Translocation into peroxisomal matrix’, translocate the enzyme from the cytosol to the matrix, across the peroxisomal membrane. Additional proteins (i.e., the members of the ubiquitin-conjugating complex Pex4p and Pex22p, as well as the members of the AAA+ complex Pex1p, Pex6p and Pex15p), which originate node 16562 ‘Peroxisomal receptor recycling’, recycle the Pex5p receptor back to the cytosol for a new round of import. Thus, given the likely sequence of events, **link 2** between docking (node 16560) and translocation (node 6625), as well as **link 3** between translocation (node 6625) and receptor recycling (node 16562), are interpreted as directional, with translocation being dependent on docking and receptor recycling on translocation.

Although perturbation of docking (node 16560), for instance upon deletion of the *pex13* gene, affects the general process of enzyme import into the peroxisome [41], it has no effect on the assembly of the translocation complex [43], thus highlighting dependence of functions (specifically, the likely dependence of translocation on docking), which is (at least in part) unrelated to the PPI among the proteins that belong to the translocation and docking complexes.

**Link 4 16560 > 16562 = Docking on peroxisomal membrane > Peroxisomal receptor recycling**

Crossing PPI: Pex13p_Pex1p; Pex13p_Pex15p; Pex13p_Pex6p

A link (**link 4**) is established between receptor recycling (node 16562) and docking (node 16560), which is interpreted as 16562 being dependent on 16560, because it is assumed that recycling requires the previous action of docking. Link 4, however, might be also regarded as non-directional, to suggest that non-directional cohesion between the docking and recycling complexes ensures tight coordination of the whole process of matrix assembly. Finally, link 4 might also be a false positive, because the shorter path between nodes 16560 and 16562 (portrayed by link 4) could be redundant with respect to the longer path (portrayed by links 2 and 3) that associates the two nodes.

The absence of a link between nodes 45184 and 16562 (with 45184 being dependent on 16562) necessitates explanation. The link was expected, because recycling into the cytosol of the Pex5p receptor (node 16562) is required for a subsequent step of enzyme recognition and binding (node 45184). The reason for the absence of the link (possibly a false negative) is twofold. First, Pex5p is not annotated in GO with the term GO:0016562 (likely because it is object, and not subject, of recycling), which prevents the algorithm from considering Pex5p as a shared protein between nodes 16562 and 45184 and thus linking the two nodes. Second, the PPI between recycling (Pex1p, Pex15p, Pex6p and Pex4p) and receptor (Pex5p) proteins have not been included as crossing PPI between nodes 16562 and 45184, because these PPI have been detected by cluster assays.

**Link 5 45046 > 16560 = PMP insertion into peroxisome membrane > Docking on peroxisomal membrane**

Crossing PPI: Pex19p_Pex13p; Pex3p_Pex13p

**Link 6 45046 > 6625 = PMP insertion into peroxisome membrane > Translocation into peroxisomal matrix**

Crossing PPI: Pex19p_Pex10p; Pex3p_Pex25p

**Link 7 45046 > 16562 = PMP insertion into peroxisome membrane > Peroxisomal receptor recycling**

Crossing PPI: Pex19p_Pex22p; Pex3p_Pex15p

Most of the proteins that mediate the steps of matrix assembly (i.e., Pex13p, Pex14p and Pex17p in node 16560; Pex2p, Pex10p and Pex12p in node 6625; Pex15p in node 16562) are PMP, which must be inserted into the peroxisome membrane for proper functioning [44]. Actually, some of these PMP establish PPI with Pex19p (and/or its membrane anchor Pex3p), which not only recognizes and binds the PMP, but also favors their insertion into the peroxisomal membrane (i.e., the process of membrane assembly). Pex19p and Pex3p belong to node 45046 ‘PMP insertion into peroxisome membrane’. Thus, the PG portrays the dependence of matrix assembly on membrane assembly, as shown by the links of node 45046 with nodes 16560, 6625 and 16562, i.e., the steps of docking (**link 5**), translocation (**link 6**) and receptor recycling (**link 7**), respectively.

**Link 8 17038 > 16560 = Protein import > Docking on peroxisomal membrane**

Crossing PPI: Pex10p_Pex13p; Pex19p_Pex13p

**Link 9 17038 > 6625 = Protein import > Translocation into peroxisomal matrix**

Crossing PPI: Pex19p_Pex12p. Shared Protein: Pex10p

**Link 10 17038 > 16562 = Protein import > Peroxisomal receptor recycling**

Crossing PPI: Pex10p_Pex1p; Pex19p_Pex22p

In addition to node 45046, node 17038 ‘Protein import’ represents a less specific type of import, which refers to the movement of proteins towards the peroxisome (with no reference to membrane insertion). By analogy with 45046, the links of node 17038 with nodes 16560, 6625 and 16562 are interpreted as directional, to indicate that docking (**link 8**), translocation (**link 9**) and receptor recycling (**link 10**) all depend on protein import. Interestingly, node 17038 annotates not only peroxisomal (Pex19p and Pex10p) but also nuclear proteins (Nup57p, Nup116p and Crm1p), which suggests a possible connection between peroxisomal and nuclear transport. The connection (albeit unexpected) is not unprecedented. For instance, the dynein Dyn2p has dual localization to peroxisomes and the nuclear pore [45].

**2. Peroxisome fission and inheritance**

Peroxisomes originate by either fission of pre-existing peroxisomes or (as discussed below in section 6) *de novo* biogenesis in the ER. During fission, peroxisomes undergo elongation and then division, to form two or more novel peroxisomes. Accordingly, node 16559 ‘Peroxisome elongation/Dnm1p-division’ comprises both elongation factors (Pex11p and Pex25p) and division factors (the dynamin Dnm1p and its partners Fis1p, Mdv1p and Caf4p). The general contribution of Pex11p to fission is based on the observation that, following exposure to oleate, yeast mutants with a disrupted pex11 gene have few and large (i.e., fission-defective) peroxisomes [46], while the more specific role of Pex11p in peroxisome elongation has been associated recently to the requirement of Pex11p for membrane curvature [47].

Related to fission is inheritance, the function whereby peroxisomes (that have duplicated by fission) distribute between mother cell and bud [48]. Inheritance is represented by node 45033 ‘Peroxisome inheritance’, which includes not only peroxisomal proteins (Pex3p, Inp1p and Inp2p) but also the non-peroxisomal motor protein Myo2p. Interestingly, the PPI internal to node 45033 clearly exemplify the way PPI contribute to function. Specifically, peroxisomes are either retained in the mother cell via the Pex3p_Inp1p PPI [49] [50] or delivered to the bud via transport along actin cables, which is mediated by Myo2. Actually, the Myo2p_Pex3p [51] and Myo2p_Inp2p PPI [52] allow peroxisomes to travel along the actin cables and to dock on the membrane of the bud. Many dependencies of peroxisome fission and inheritance are portrayed in the graph, as discussed below.

**Link 11 45046 > 16559 = PMP insertion into peroxisome membrane > Peroxisome elongation/Dnm1p-division**

Crossing PPI: Pex19p_Pex11p; Pex3p_Pex11p

**Link 12 45046 > 45033 = PMP insertion into peroxisome membrane > Peroxisome inheritance**

Crossing PPI: Pex19p_Inp2p. Shared Protein: Pex3p

First, like many mediators of matrix assembly, also some mediators of fission (Pex11p and Pex25p) and inheritance (Inp1p and Inp2p) are PMP, which require Pex19p and Pex3p for proper localization. Thus, both ‘Peroxisome elongation/Dnm1p-division’ (node 16559) and ‘Peroxisome inheritance’ (node 45033) depend on ‘PMP insertion into peroxisome membrane’ (node 45046), as portrayed in **link 11** and **link 12**, respectively. Note that link 12 is also based on the sharing of protein Pex3p, even though the inheritance- and PMP insertion-related pools of Pex3p might be distinct [50].

**Link 13 16559 > 45033 = Peroxisome elongation/Dnm1p-division > Peroxisome inheritance**

Crossing PPI: Pex25p_Inp1p; Pex11p_Pex3p

Second, **link 13** highlights the dependence of ‘Peroxisome inheritance’ (node 45033) on ‘Peroxisome elongation/Dnm1p-division’ (node 16559), which is based on the finding that, following exposure to oleate, peroxisomes are absent from most of the buds in Pex11p-deficient yeasts. Possibly, defective fission (due to Pex11p absence) results in the formation of giant peroxisomes that are difficult to segregate from the mother cell to the bud [46].

**Link 14 266 > 16559 = Regulation of Dnm1p > Peroxisome elongation/Dnm1p-division**

Crossing PPI: Rpn11p_Dnm1p. Shared Proteins: Caf4p;Fis1p;Mdv1p

**Link 15 266 > 1 = Regulation of Dnm1p > Mitochondrion Dnm1p-division**

Crossing PPI: Caf4p_Dnm1p; Fis1p_Dnm1p; Mdv1p_Dnm1p; Rpn11p_Dnm1p

Third, in addition to peroxisome fission, the Dnm1p-based division is also required for mitochondrion fission [53]. Specifically, node 1 ‘Mitochondrion Dnm1p-division’ annotates Dnm1p and its associated protein Num1p. Furthermore, the Dnm1p-dependent division of both peroxisomes (node 16559) and mitochondria (node 1) is positively regulated by the metalloprotease Rpn11p [54], which belongs to node 266 ‘Regulation of Dnm1p’. Although Rpn11p mediates substrate de-ubiquitination and proteasomal degradation, the role of Rpn11p in organelle fission is unrelated to its role in the proteasome [54]. The dependence of the Dnm1p-mediated fission of peroxisomes and mitochondria on Rpn11p is shown by **link 14** (between nodes 266 and 16559) and **link 15** (between nodes 266 and 1), respectively.

**Link 16 16559 > 70584 = Peroxisome elongation/Dnm1p-division > Peroxisome Vps1p-division**

Crossing PPI: Dnm1p_Rpn11p

**Link 17 1 > 70584 = Mitochondrion Dnm1p-division > Peroxisome Vps1p-division**

Crossing PPI: Dnm1p_Rpn11p

Fourth, besides Dnm1p, an additional (peroxisome-specific) division machinery relies on the dynamin Vps1p [55], as represented by node 70584 ‘Peroxisome Vps1p-division’. Although the Vps1p and Dnm1p systems are autonomous, **link 16** suggests that the Vps1p system (node 70584) may depend on the Dnm1p system (node 16559), because also the Vps1p-based division of peroxisomes requires the elongation step, which is included in node 16559. Furthermore, **link 17** suggests that the Vps1p-based fission of peroxisomes (node 70584) may also depend on the Dnm1p-based fission of mitochondria (node 1).

Increased production of peroxisomes in response to mitochondrial dysfunction (e.g., impaired respiration in glucose) reinforces the suggestion of link 17. Actually, mitochondrion and peroxisome fission are coordinated, because the peroxisomal Vps1p machinery is activated when the Dnm1p-based mitochondrial system fails [56].

**Link 18 16559 > 1300 = Peroxisome elongation/Dnm1p-division > Cell aging**

Crossing PPI: (none). Shared Proteins: Dnm1p; Fis1p

**Link 19 70584 > 1300 = Peroxisome Vps1p-division > Cell aging**

Crossing PPI: Rpn11p_Dnm1p

**Link 20 266 > 1300 = Regulation of Dnm1p > Cell aging**

Crossing PPI: Caf4p_Dnm1p. Shared Protein: Fis1p

Fifth, node 1300 ‘Cell aging’ depends on the Dnm1p- (node 16559) (**link 18**) and Vps1p-mediated fission (node 70584) (**link 19**) of peroxisomes, as well as on the Rpn11p-mediated regulation of Dnm1p (node 266) (**link 20**). The peroxisomal proteins in node 1300 (Dnm1p and Fis1p) are associated with aging, because Dnm1p binds the histone deacetylase Sir2p and because mutation of either Dnm1p or Fis1p affects life span or apoptosis. For instance, null mutation of dnm1 increases life span [57]. Thus, life span might be associated not only with mitochondria, but also with peroxisomes.

**Link 21 17038 > 16559 = Protein import > Peroxisome elongation/Dnm1p-division**

Crossing PPI: Hsp82p_Pex11p; Nup11p_Mdv1p; Pex10p_Pex11p; Pex19p_Pex11p

**Link 22 17038 > 70584 = Protein import > Peroxisome Vps1p-division**

Crossing PPI: Crm1p_Rpn11p; Pex19p_Vps1p

**Link 23 17038 > 266 = Protein import > Regulation of Dnm1p**

Crossing PPI: Crm1p_Rpn11p; Hsp82p_Rpn11p; Nup11p_Mdv1p

**Link 24 17038 > 45033 = Protein import > Peroxisome inheritance**

Crossing PPI: Pex19p_Inp2p; Crm1p_Myo2p; Pex19p_Pex3p

Sixth, additional links indicate the dependence on protein import (node 17038) of the Dnm1p-mediated (node 16559) (**link 21**) and Vps1p-mediated (node 70584) (**link 22**) fission of peroxisomes, as well as the regulation of Dnm1p (node 266) (**link 23**) and the inheritance of peroxisomes (node 45033) (**link 24**). Particularly important for peroxisome fission is the Pex19p_Vps1p PPI underlying link 22, because mutants (lacking either the vps1 gene or, more importanly, the Pex19p recognition sequence of Vps1p) produce few and large peroxisomes. It is more likely that the effect of Pex19p is to target Vps1p to the peroxisomes than to insert Vps1p into the peroxisomal membrane [58]. In any case, the PG correctly indicates the two different ways, whereby Pex19p contributes to peroxisome fission. Specifically, while link 11 indicates that Pex19p inserts the elongation factor Pex11p into the peroxisomal membrane, link 22 indicates that Pex19p targets the division factor Vps1p to the peroxisome.

**Link 25 16559 > 16560 = Peroxisome elongation/Dnm1p-division > Docking on peroxisomal membrane**

Crossing PPI: Pex11p_Pex13p; Pex11p_Pex14p

**Link 26 16559 > 6625 = Peroxisome elongation/Dnm1p-division > Translocation into peroxisomal matrix**

Crossing PPI: Pex11p_Pex10p. Shared Protein: Pex25p

**Link 27 16559 > 16562 = Peroxisome elongation/Dnm1p-division > Peroxisomal receptor recycling**

Crossing PPI: Pex11p_Pex15p; Pex11p_Pex4p

**Link 28 45033 > 16560 = Peroxisome inheritance > Docking on peroxisomal membrane**

Crossing PPI: Myo2p_Pex7p; Pex3p_Pex13p

**Link 29 45033 > 6625 = Peroxisome inheritance > Translocation into peroxisomal matrix**

Crossing PPI: Inp1p_Pex25p; Pex3p_Pex25p

Finally, other links suggest the dependence of matrix assembly-related functions (nodes 16560, 6625 and 16562) on biogenesis-related functions, such as fission and inheritance (nodes 16559 and 45033). However, the PPI underlying the links of node 16559 with nodes 16560 (**link 25**), 6625 (**link 26**) and 16562 (**link 27**) mightr not be direct PPI, as they are derived from protein complementation assays [59]. Furthermore, some of the PPI underlying the link of inheritance (node 45033) with nodes 16560 (**link 28**) (Pex3p_Pex13p) and 6625 (**link 29**) (Pex3p_Pex25p) rely on the inclusion of Pex3p in node 45033. Thus, links 28 and 29 might be false positives, as discussed in the main text.

**3. Regulation of peroxisomal protein localization**

Many links in the PG refer to the regulation of protein localization, as detailed below.

**Link 30 32880 > 16559 = Regulation of protein localization > Peroxisome elongation/Dnm1p-division**

Crossing PPI: Pho85p_Pex11p; Rho1p_Pex25p

**Link 31 32880 > 6625 = Regulation of protein localization > Translocation into peroxisomal matrix**

Crossing PPI: Pho85p_Pex10p; Rho1p_Pex25p

First, **link 30** between ‘Regulation of protein localization’ (node 32880) and ‘Peroxisome elongation/Dnm1p-division’ (node 16559) indicates that peroxisome fission depends on regulatory mechanisms of protein localization. Specifically, link 30 relies on the Pho85p-dependent phosphorylation of Pex11p, which induces the fission effector Pex11p to change location from the ER to the peroxisome [27]. Similarly, **link 31** suggests that also translocation into the matrix (node 6625) depends on a similar regulation, because Pho85p (node 32880) also phosphorylates Pex10p (node 6625), even though the purported dependence needs experimental confirmation. Notably, the second PPI of link 30 (Rho1p_Pex25p) indicates that the fission effector Pex25p binds the GTPase Rho1p, thus possibly linking fission with actin assembly [60]. Pex25p has been also involved in peroxisome formation in peroxisome-deficient cells [61].

**Link 32 32880 > 266 = Regulation of protein localization > Regulation of Dnm1p**

Crossing PPI: Pho85p_Rpn11p; Rho1p_Rpn11p; Rsp5p_Rpn11p

Second, **link 32** indicates that also ‘Regulation of Dnm1p’ (node 266) depends on node 32880. Thus, the above mentioned dependence of ‘Peroxisome elongation/Dnm1p-division’ (node 16559) on node 266 (link 14) raises the question, whether the direct link between nodes 32880 and 16559 (link 30) is redundant with respect to the path that connects nodes 32880 and 266 (link 32) and then nodes 266 and 16559 (link 14). However, this is not likely to be the case, because links 30 and 14 have different targets (the elongation factor Pex11p and the division factor Dnm1p, respectively).

It should be noted that the types of evidence supporting the two links of the path (i.e., links 32 and 14) have different strength. Concerning link 14, Rpn11p (in node 266) was demonstrated to activate Dnm1p (in node 16559) and thus to induce peroxisome fission. In contrast, concerning link 32, the putative induction of Rpn11p localization (e.g., from the proteasome) to the peroxisome is just inferred from PPI (Pho85p_Rpn11p, Rsp5p_Rpn11p and Rho1p_Rpn11p) that are derived from an affinity-capture screen [54] and that therefore do not necessarily imply post-translational modifications of Rpn11p.

**Link 33 32880 > 48856 = Regulation of protein localization > Cortical actin dynamics**

Crossing PPI: Rsp5p_Sla1p; Rho1p_Ubi4p

**Link 34 48856 > 16559 = Cortical actin dynamics > Peroxisome elongation/Dnm1p-division**

Crossing PPI: Ubi4p_Dnm1p; Yak1p_Dnm1p

**Link 35 48856 > 1 = Cortical actin dynamics > Mitochondrion Dnm1p-division**

Crossing PPI: Ubi4p_Dnm1p; Yak1p_Dnm1p

**Link 36 48856 > 1300 = Cortical actin dynamics > Cell aging**

Crossing PPI: Ubi4p_Dnm1p; Yak1p_Dnm1p

**Link 37 48856 > 45033 = Cortical actin dynamics > Peroxisome inheritance**

Crossing PPI: Ubi4p_Myo2p; Vps1p_Inp1p

**Link 38 17038 > 48856 = Protein import > Cortical actin dynamics**

Crossing PPI: Crm1p_Ubi4p; Pex19p_Vps1p

Third, **link 33** indicates the dependence of node 48856 ‘Cortical actin dynamics’ on node 32880. In turn, link 34 indicates the dependence of ‘Peroxisome elongation/Dnm1p-division’ (node 16559) on node 48856. As above, link 30 (between nodes 32880 and 16559) is not redundant with respect to the path that connects nodes 32880 and 48856 (**link 33**) and then nodes 48856 and 16559 (**link 34**), because links 30 and 34 have different targets (Pex11p and Dnm1p, respectively). Furthermore, like node 16559, also mitochondrion fission (node 1) (**link 35**), cell aging (node 1300) (**link 36**) and peroxisome inheritance (node 45033) (**link 37**) depend on cortical actin dynamics (node 48856). Importantly, the PPI Pex19p_Vps1p, which links protein import (node 17038) and node 48856 (**link 38**), may connect peroxisomes with the peculiar activities of Vps1p that are portrayed in node 48856, as detailed here.

Within node 48856, a set of PPI centered on Vps1p associate actin remodeling and ubiquitination. In particular, the Vps1p_Sla1p PPI recruits the actin-binding protein Sla1p to sites of actin remodeling and endocytosis [62]. In addition, the Ubi4p_Vps1p and Ubi4p_Sla1p PPI indicate that Vps1p and Sla1p can be ubiquitinated. A likely effector is the E3 ubiquitin-ligase Rsp5p [63], which belongs to node 32880. In general, Rsp5p poly-ubiquitinates substrates that must be targeted from the endosome to the vacuole for degradation [64]. However, the Rsp5p-induced ubiquitination of link 33 does not likely refer to poly-ubiquitination of substrates that must be degraded, but rather to mono-ubiquitination of proteins (mostly SH3 proteins like Sla1p) that modulate endocytosis. Rsp5p was indeed shown to regulate actin organization and endocytosis via mono-ubiquitination of Sla1p and its partner Rvs167p [65], two proteins of the actin cortical patch, which is an endocytosis-related structure of the plasma membrane. In addition, the Rsp5p effect indeed refers to the regulation of protein localization, as Rsp5p targets Sla1p to the cortex [66]. The functional connection of cortical actin and endocytosis (node 48856) with the fission of peroxisomes (node 16559) and mitochondria (node 1), however, remains less defined, even though it may involve membrane modifications. For instance, Vps1p and Rvs167p (and its partner Rvs161p) mediate vesicle fission and fusion, by inducing different types of membrane invagination, including the assembly of tubular structures that are made up of lipids and proteins [67, 68].

In summary, by localizing Sla1p to sites of actin remodeling, Rsp5p (node 32880) may facilitate, in an ubiquitin-dependent way, the Vps1p-regulated development of membrane structures (node 48856), which (similarly to endocytosis) in turn facilitate peroxisome (and mitochondrion) fission. Noteworthy, a recent study has confirmed the importance of ubiquitination in peroxisome biogenesis [69].

**4. Regulation of peroxisomal protein expression**

In addition to regulating protein localization, other processes may regulate protein levels, by controlling either synthesis or degradation. Concerning synthesis, carbon source-responsive transcription factors (e.g., Adr1p, Oaf1p and Pip2p) bind and activate regulatory elements of peroxisomal genes. However, synthesis regulation does not primarily rely on PPI, but on protein-DNA interactions (i.e., on interactions between transcription factors and gene regulatory elements), which are not represented in a PPI network. Concerning stability, the following links suggest possible mechenisms.

**Link 39 19538 > 16560 = Protein folding > Docking on peroxisomal membrane**

Crossing PPI: Cct2p_Pex7p; Cct3p_Pex7p; Cct5p_Pex7p; Cct6p_Pex7p

**Link 40 19538 > 16559 = Protein folding > Peroxisome elongation/Dnm1p-division**

Crossing PPI: Cct2p_Caf4p; Cct3p_Caf4p; Cct5p_Caf4p; Cct6p_Caf4p

**Link 41 19538 > 266 = Protein folding > Regulation of Dnm1p**

Crossing PPI: Cct2p_Caf4p; Cct3p_Caf4p; Cct5p_Caf4p; Cct6p_Caf4p

Node 19538 ‘Protein folding’ annotates proteins of the chaperonin-containing T-complex, which mediates protein folding in the cytosol. Thus, the graph suggests that folding may regulate the stability of the proteins that are involved in matrix import (node 16560) (**link 39**) and fission (nodes 16559 and 266) (**link 40** and **link 41**), even though experimental support is missing.

**5. Other functional features of peroxisome biology: interactions among metabolic processes**

**Link 42 45046 > 19395 = PMP insertion into peroxisome membrane > Fatty acid oxidation**

Crossing PPI: Pex19p_Pex11p; Pex3p_Pex11p

**Link 43 17038 > 19395 = Protein import > Fatty acid oxidation**

Crossing PPI: Hsp82p_Ant1p; Hsp82p_Mdh3p; Hsp82p_Pex11p

**Link 44 32880 > 19395 = Regulation of protein localization > Fatty acid oxidation**

Crossing PPI: Pho85p_Fox2p; Rsp5p_Fox2p

The PG highlights metabolic processes of the peroxisome. In particular, **link 42** indicates the dependence of node 19395 ‘Fatty acid oxidation’ on the Pex19p-mediated insertion (node 45046) of the PMP Pex11p (node 19395) into the peroxisome membrane. In this location, Pex11p provides fatty acid oxidases of the peroxisomal matrix (e.g., Faa2p) with substrates [70]. In addition, fatty acid oxidation (node 19395) depends on other less characterized processes, such as protein import (node 17038) (**link 43**) or localization regulation (node 32880) (**link 44**).

**Link 45 5977 > 19395 = Glycogen biosynthesis inhibition > Fatty acid oxidation**

Crossing PPI: Pho85p_Fox2p; Pho85p_Pex11p

**Link 46 5977 > 266 = Glycogen biosynthesis inhibition > Regulation of Dnm1p**

Crossing PPI: Pho85p_Rpn11p; Reg1p_Rpn11p

Node 5977 ‘Glycogen biosynthesis inhibition’ provides an intriguing example of relations between metabolic and biogenetic processes. The node annotates Pho85p and Reg1p, both of which prevent glycogen biosynthesis in response to glucose availability. Specifically, Pho85p (together with the Pcl8p and Pcl10p cyclins) inhibits (by phosphorylation) the glycogen synthase Gsy2p [71]. Similarly, Reg1p (together with the catalytic phosphatase subunit Glc7p) inhibits (by de-phosphorylation) the activator of glucose-repressed genes Snf1p.

**Link 45** and **link 46** suggest that fatty acid oxidation (node 19395) and the Rpn11p-dependent regulation of the fission regulator Dnm1p (node 266) depend on glycogen biosynthesis inhibition (node 5977). The directionality of these two links is just inferred from the biochemical directionality of the underlying PPI. Nevertheless, if experimentally proven, these functional links might highlight coordination of peroxisome fission, fatty acid oxidation and glycogen biosynthesis in response to glucose availability. One might speculate that increased glucose reduces the need not only for glycogen accumulation but also for peroxisome proliferation and fatty acid oxidation.

As a general comment, it should be noted that FN representing metabolic functions are under-represented in the PG. The plausible explanation is that, while the PG is based on PPI, the metabolic enzymes seldom interact physically by means of PPI. More often, enzymes interact functionally by means of shared chemical compounds (typically, a compound that is product and substrate of two adjacent enzymes in a metabolic pathway). Actually, metabolic maps are a subtype of interactome networks, in which nodes and edges represent enzymes and shared compounds, respectively [72]. Thus, typical metabolic functions of the peroxisomes, i.e., fatty acid beta-oxidation and oxygen radical scavenging, are not adequately represented in the PG. Specifically, peroxisomal proteins of the PPI network (Ant1p, Eci1p, Fox2p, Idp3p, Mdh3p, Pot1p, Pox1p and Tes1p), albeit annotated with the term ‘Fatty acid beta oxidation’ (GO:0006635), do not interact mutually by means of PPI, but only by means of shared compounds (e.g., trans-2-enoyl-CoA, which is product of Pox1p and substrate of Fox2p), so that no node 6635 is generated. In addition, even if node 6635 were generated, it might not easily establish links with other metabolic nodes. For instance, oxygen radical scavenging depends on fatty acid beta-oxidation, because oxidation is the major source of hydrogen peroxide in the peroxisome. Yet, the peroxisomal catalase Cta1p is not linked with any of the beta-oxidation enzymes by means of PPI, but only by means of shared compounds. Specifically, the fatty acid oxidase Fox2p and the catalase Cta1p are linked by the shared compound hydrogen peroxide, which is product of Fox2p and substrate of Cta1p. Thus, FN related to fatty acid beta-oxidation and radical scavenging are not linked mutually in the PG.

**6. ER-dependent regulation of peroxisome biogenesis**

Usually peroxisomes derive by fission of pre-existing peroxisomes [73], even though, when peroxisomes are absent (for instance, in pex3 or pex19 mutants), peroxisomes can be generated de novo from the ER [74]. Recent evidence, however, indicates that the ER contributes to not only to the de novo biogenesis of peroxisomes but also to their fission. Yet, as knowledge about the molecular basis of these processes is incomplete, node 32581, which represents the ‘ER-dependent peroxisome biogenesis’, lacks direct links with the other FN of the peroxisomal core, including the fission-related FN. The lack of connections is attribuatble to annotation defects, because de novo biogenesis shares Pex3p (and Pex19p) with key peroxisome-based FN (e.g., the PMP insertion node 45046). In addition, Pex3p localizes in small dot-like structures of the ER that bud off (in a Pex19p-dependent manner) to form Pex3p-containing vesicles, which in turn fuse with fission-derived peroxisomes. In this way, the ER transfers membranes [20] and PMP [75] to the growing peroxisomes. However, the link between nodes 45046 and 32581 is absent from the PG, because Pex3p and Pex19p were not included in term 32581.

**Part II: List of FN**

**FN 45184**

*Annotated GO term(s)*: 45184- 55 - 51169 - 54 - 6623 - 6997 - 15031 - 32581 - 6913 - 51170 - 6607 - 34504 - 6407 - 50658 - 6611 - 51168

*Original label:* Establishment of protein localization-[Ribosomal large subunit export from nucleus]-[Nuclear transport]-[Ribosomal subunit export from nucleus]-[Protein targeting to vacuole]-[Nucleus organization]-[Protein transport]-[ER-dependent peroxisome biogenesis]-[Nucleocytoplasmic transport]-[Nuclear import]-[NLS-bearing substrate import into nucleus + snRNP protein import into nucleus + mRNA-binding (hnRNP) protein import into nucleus + Ribosomal protein import into nucleus]-[Protein localization to nucleus]-[rRNA export from nucleus]-[RNA transport]-[Protein export from nucleus]-[Nuclear export]

*New label*: Peroxisomal receptor-enzyme recognition

*Protein content*: Pex5p

*Notes*: The FN has been relabeled ‘Peroxisomal receptor-enzyme recognition’, because (after undergoing numerous enucleation procedures) its protein content has been reduced to the peroxisomal receptor Pex5p, which recognizes the metabolic enzymes that must be transported from the cytosol into the peroxisomal matrix. In accordance with the definition of the original label (‘directed movement of a protein to a specific location’), the recognition of these enzymes is the first step leading to their specific localization to the peroxisome.

**FN 16560**

*Annotated GO term(s)*: 16560- 45184

*Original label:* Protein import into peroxisome matrix, docking -[see above, FN 45184]

*New label*: Docking on peroxisomal membrane

*Protein content*: Pex13p, Pex14p, Pex17p, Pex7p

*Notes*: The FN label has undergone a minor change.

**FN 6625**

*Annotated GO term(s)*: 6625- 45046

*Original label:* Protein targeting to peroxisome -[Protein import into peroxisome membrane]

*New label*: Translocation into peroxisomal matrix

*Protein content*: Pex10p, Pex12p, Pex2p, Pex22p, Pex25p, Pex8p

*Notes*: As mentioned in the main text (and as shown in Figure 2D), FN 6625 exemplifies the relabeling procedure that follows an enucleation step. In particular, the initial annotation of FN 6625, which is based on term GO:0006625, refers rather generically to the targeting of proteins to the peroxisomes. However, part of the protein content of FN 6625 (Pex3p and Pex19p), which matches the content of the FN annotated with term GO:0045046, refers more specifically to the assembly of the peroxisomal membrane. Thus, the function of membrane assembly is enucleated from node 6625 and retained in node 45046. As node 6625 now excludes the enucleated function, its residual content in proteins (in particular, the linker Pex8p and the members of the RING finger complex Pex2p, Pex10p and Pex12p) more specifically refers to the targeting to the peroxisome that requires translocation into peroxisomal matrix. The FN has been renamed accordingly.

**FN 16562**

*Annotated GO term(s)*: 16562

*Original label:* Protein import into peroxisome matrix, receptor recycling

*New label*: Peroxisomal matrix, receptor recycling

*Protein content*: Pex1p, Pex15p, Pex22p, Pex4p, Pex6p

*Notes*: The FN label has undergone a minor change.

**FN 45046**

*Annotated GO term(s)*: 45046

*Original label:* Protein import into peroxisome membrane

*New label*: PMP insertion into peroxisome membrane

*Protein content*: Pex19p, Pex3p

*Notes*: The FN label has undergone a minor change, to denote that the specific protein targets of Pex19p and Pex3p action are the PMP.

**FN 17038**

*Annotated GO term(s)*: 17038

*Original label:* Protein import

*New label*: Protein import

*Protein content*: Crm1p, Hsp82p, Nup116p, Nup57p, Pex10p, Pex19p

*Notes*: The original FN label has not been changed.

**FN 16559**

*Annotated GO term(s)*: 16559- 70584

*Original label:* Peroxisome fission -[Mitochondrion morphogenesis]

*New label*: Peroxisome elongation/Dnm1p-division

*Protein content*: Caf4p, Dnm1p, Fis1p, Mdv1p, Pex11p, Pex25p

*Notes*: Node 16559 has been labeled ‘Peroxisome elongation/Dnm1p-division’, because (after enucleation of Rpn11p and Vps1p) it comprises both elongation factors (Pex11p and Pex25p) and division factors (the dynamin Dnm1p and its partners Fis1p, Mdv1p and Caf4p) that are both involved in the Dnm1p-dependent fission of peroxisomes.

**FN 45033**

*Annotated GO term(s)*: 45033

*Original label:* Peroxisome inheritance

*New label*: Peroxisome inheritance

*Protein content*: Inp1p, Inp2p, Myo2p, Pex3p

*Notes*: The original FN label has not been changed.

**FN 266**

*Annotated GO term(s)*: 266- 1

*Original label:* Mitochondrial fission -[Mitochondrion inheritance]

*New label*: Regulation of Dnm1p

*Protein content*: Caf4p, Fis1p, Mdv1p, Rpn11p

*Notes*: FN 266 has been labeled ‘Regulation of Dnm1p’, because the metalloprotease Rpn11p positively regulates the Dnm1p-dependent division of both peroxisomes (FN 16559) and mitochondria (FN 1).

**FN 1**

*Annotated GO term(s)*: 1

*Original label:* Mitochondrion inheritance

*New label*: Mitochondrion Dnm1p-division

*Protein content*: Dnm1p, Num1p

*Notes*: FN 1 has been labeled ‘Mitochondrion Dnm1p-division’, because it contains Dnm1p and its associated protein Num1p, which mediate mitochondrion fission.

**FN 70584**

*Annotated GO term(s)*: 70584

*Original label:* Mitochondrion morphogenesis

*New label*: Peroxisome Vps1p-division

*Protein content*: Rpn11p, Vps1p

*Notes*: FN 70584 has been relabeled ‘Peroxisome Vps1p-division’, because it contains the dynamin Vps1p, which represents an additional (and peroxisome-specific) division system (different from the Dnm1p-based system, which is shared with mitochondria and is represented by FN 16559).

**FN 1300**

*Annotated GO term(s)*: 1300

*Original label:* Chronological cell aging

*New label*: Cell aging

*Protein content*: Dnm1p, Fis1p, Sir2p

*Notes*: The FN label has undergone a minor change (for brevity’s sake).

**FN 32880**

*Annotated GO term(s)*: 32880

*Original label:* Regulation of protein localization

*New label*: Regulation of protein localization

*Protein content*: Bni1p, Pho85p, Rho1p, Rsp5p, Zds2p

*Notes*: The original FN label has not been changed.

**FN 48856**

*Annotated GO term(s)*: 48856

*Original label:* Anatomical structure development

*New label*: Cortical actin dynamics

*Protein content*: Rsc2p, Sla1p, Ubi4p, Vps1p, Yak1p

*Notes*: The reasons that led to renaming FN 48856 as ‘Cortical actin dynamics’ have been detailed above. Briefly, a subset of proteins within the node associate remodeling of cortical actin (Vps1p and Sla1p) with ubiquitination (Ubi4p and Rsp5p). The consistency with the definition of the original GO term (‘… progression of an anatomical structure from an initial condition to its mature state’) is partially ensured by the association with the development of membrane structures, which may facilitate peroxisome (and mitochondrion) fission, as explained above.

**FN 19538**

*Annotated GO term(s)*: 19538

*Original label:* Protein metabolic process

*New label*: Protein folding

*Protein content*: Cct2p, Cct3p, Cct5p, Cct6p, Cdc20p

*Notes*: FN 19538 has been labeled ‘Protein folding’, because the node provides just a minor coverage of the *S. cerevisiae* proteins annotated by the GO:0019538 term ‘Protein metabolic process’ (5 out of 1,740 proteins). The FN has been first relabeled ‘Protein modification’, to adapt the label to the actual function of its content. In particular, 4 proteins (the T-complex members Cct2p, Cct3p, Cct5p and Cct6p) mediate protein folding, while 1 protein (the APC complex activator Cdc20p) mediates protein catabolism. However, the node has been further relabeled ‘Protein folding’, to adapt the label to the relations of FN 19538 within the peroxisome PG, because all the links of FN19538 are mediated by the T-complex proteins. In any case, the label is consistent with the definition of the initial GO-based label (‘… the chemical reactions involving a specific protein … includes protein modification.’), because protein folding is indeed a set of chemical reactions that causes protein modification.

**FN 19395**

*Annotated GO term(s)*: 19395

*Original label:* Fatty acid oxidation

*New label*: Fatty acid oxidation

*Protein content*: Ant1p, Fox2p, Mdh3p, Pex11p

*Notes*: The original FN label has not been changed.

**FN 5977**

*Annotated GO term(s)*: 5977

*Original label:* Glycogen metabolic process

*New label*: Glycogen biosynthesis inhibition

*Protein content*: Pho85p, Reg1p

*Notes*: The FN label has undergone a minor change, to signify that the protein content of the node (Pho85p and Reg1p) specifically regulate glycogen biosynthesis.

**FN 32581**

*Annotated GO term(s)*: 32581

*Original label:* ER-dependent peroxisome biogenesis

*New label*: ER-dependent peroxisome biogenesis

*Protein content*: Dsl1p, Sec20p, Sec39p

*Notes*: The original FN label has not been changed.

**Part III: Inference of edge directions**

**Link 1**

*A>B*: 45184 > 16560 (Peroxisomal receptor-enzyme recognition > Docking on peroxisomal membrane)

*Inference rule*: Domain knowledge (event A precedes event B; rule 4)

*Note*: Enzyme recognition in the cytosol precedes docking onto the peroxisomal membrane.

**Link 2**

*A>B*: 16560 > 6625 (Docking on peroxisomal membrane > Translocation into peroxisomal matrix)

*Inference rule*: Domain knowledge (event A precedes event B; rule 4)

*Note*: Docking onto the peroxisomal membrane precedes translocation across the same membrane.

**Link 3**

*A>B*: 6625 > 16562 (Translocation into peroxisomal matrix > Peroxisomal receptor recycling)

*Inference rule*: Domain knowledge (event A precedes event B; rule 4)

*Note*: Receptor translocation into the peroxisomal matrix precedes recycling out of the matrix.

**Link 4**

*A>B*: 16560 > 16562 (Docking on peroxisomal membrane > Peroxisomal receptor recycling)

*Inference rule*: Domain knowledge (event A precedes event B; rule 4)

*Note*: Dubious edge, possibly a false positive (see above). In any case, docking precedes recycling.

**Link 5**

*A>B*: 45046 > 16560 (PMP insertion into peroxisomal membrane > Docking on peroxisomal membrane)

*Inference rule*: Domain knowledge (B logically implies A; rule 3)

*Note*: Some peroxisomal docking proteins are PMP that require being inserted in the peroxisomal membrane for proper functioning. In other terms, 16560 cannot be true, unless 45046 is true.

**Link 6**

*A>B*: 45046 > 6625 (PMP insertion into peroxisomal membrane > Translocation into peroxisomal matrix)

*Inference rule*: Domain knowledge (B logically implies A; rule 3)

*Note*: Some peroxisomal translocation proteins are PMP that require being inserted in the peroxisomal membrane for proper functioning.

**Link 7**

*A>B*: 45046 > 16562 (PMP insertion into peroxisomal membrane > Peroxisomal receptor recycling)

*Inference rule*: Domain knowledge (B logically implies A; rule 3)

*Note*: Some peroxisomal recycling proteins are PMP that require being inserted in the peroxisomal membrane for proper functioning.

**Link 8**

*A>B*: 17038 > 16560 (Protein import > Docking on peroxisomal membrane)

*Inference rule*: Domain knowledge (B logically implies A; rule 3)

*Note*: Docking proteins require being imported to the peroxisome for proper functioning.

**Link 9**

*A>B*: 17038 > 6625 (Protein import > Translocation into peroxisomal matrix)

*Inference rule*: Domain knowledge (B logically implies A; rule 3)

*Note*: Translocation proteins require being imported to the peroxisome for proper functioning.

**Link 10**

*A>B*: 17038 > 16562 (Protein import > Peroxisomal receptor recycling)

*Inference rule*: Domain knowledge (B logically implies A; rule 3)

*Note*: Recycling proteins require being imported to the peroxisome for proper functioning.

**Link 11**

*A>B*: 45046 > 16559 (PMP insertion into peroxisomal membrane > Peroxisome elongation/Dnm1p-division)

*Inference rule*: Domain knowledge (B logically implies A; rule 3)

*Note*: Some fission proteins are PMP that require being inserted in the peroxisomal membrane for proper functioning.

**Link 12**

*A>B*: 45046 > 45033 (PMP insertion into peroxisomal membrane > Peroxisome inheritance)

*Inference rule*: Domain knowledge (B logically implies A; rule 3)

*Note*: Some inheritance proteins are PMP that require being inserted in the peroxisomal membrane for proper functioning.

**Link 13**

*A>B*: 16559 > 45033 (Peroxisome elongation/Dnm1p-division > Peroxisome inheritance)

*Inference rule*: Experimental evidence (manipulation of A affects B; rule 1)

*Note*: The statement that manipulation of A (fission) affects B (inheritance) is based on the experimental evidence that deficiency of the fission factor Pex11p results not only in the presence of giant (i.e., non-divided) peroxisomes in the mother cell (i.e., deficiency of fission), but also in the absence of peroxisomes in the bud cell (i.e., deficiency of inheritance).

**Link 14**

*A>B*: 266 > 16559 (Regulation of Dnm1p > Peroxisome elongation/Dnm1p-division)

*Inference rule*: Experimental evidence (manipulation of A affects B; rule 1)

*Note*: Experimental evidence indicates that defective Rpn11p function results in deficiency of the Dnm1p-dependent division (fission) of peroxisomes.

**Link 15**

*A>B*: 266 > 1 (Regulation of Dnm1p > Mitochondrion Dnm1p-division)

*Inference rule*: Experimental evidence (manipulation of A affects B; rule 1)

*Note*: Experimental evidence indicates that defective Rpn11p function results in deficiency of the Dnm1p-dependent division (fission) of mitochondria.

**Link 16**

*A>B*: 16559 > 70584 (Peroxisome elongation/Dnm1p-division > Peroxisome Vps1p-division)

*Inference rule*: Domain knowledge (event A precedes event B; rule 4)

*Note*: Peroxisome elongation precedes the Vps1p-dependent division (fission) of peroxisomes.

**Link 17**

*A>B*: 1 > 70584 (Mitochondrion Dnm1p-division > Peroxisome Vps1p-division)

*Inference rule*: Experimental evidence (manipulation of A affects B; rule 1)

*Note*: Experimental evidence indicates that failure of the Dnm1p-dependent division (fission) of mitochondria results in the activation of the Vps1p-dependent division (fission) of peroxisomes.

**Link 18**

*A>B*: 16559 > 1300 (Peroxisome elongation/Dnm1p-division > Cell aging)

*Inference rule*: Biological knowledge (A might influence B; rule 6)

*Note*: General biological knowledge suggests that oxidative metabolism (like the one occurring in peroxisomes) is known to affect cell aging. Thus, Dnm1p-dependent fission and formation of new peroxisomes might influence aging.

**Link 19**

*A>B*: 70584 > 1300 (Peroxisome Vps1p-division > Cell aging)

*Inference rule*: Biological knowledge (A might influence B; rule 6)

*Note*: General biological knowledge suggests that oxidative metabolism (like the one occurring in peroxisomes) is known to affect cell aging. Thus, Vps1p-dependent fission and formation of new peroxisomes might influence aging.

**Link 20**

*A>B*: 266 > 1300 (Regulation of Dnm1p > Cell aging)

*Inference rule*: Biological knowledge (A might influence B; rule 6)

*Note*: General biological knowledge suggests that oxidative metabolism (like the one occurring in peroxisomes and mitochondria) is known to affect cell aging. Thus, Dnm1p-dependent fission and formation of new peroxisomes and mitochondria might influence aging.

**Link 21**

*A>B*: 17038 > 16559 (Protein import > Peroxisome elongation/Dnm1p-division)

*Inference rule*: Domain knowledge (event A precedes event B; rule 4)

*Note*: Importing fission proteins to the peroxisome precedes peroxisome fission.

**Link 22**

*A>B*: 17038 > 70584 (Protein import > Peroxisome Vps1p-division)

*Inference rule*: Experimental evidence (manipulation of A affects B; rule 1)

*Note*: The statement that manipulation of A (protein import) affects B (Vps1p-dependent fission of peroxisomes) is based on the experimental evidence that preventing the fission factor Vps1p from binding the peroxisomal importer Pex19p results in the formation of fission-defective peroxisomes.

**Link 23**

*A>B*: 17038 > 266 (Protein import > Regulation of Dnm1p)

*Inference rule*: Domain knowledge (event A precedes event B; rule 4)

*Note*: Importing fission regulators to the peroxisome precedes regulation of peroxisome fission.

**Link 24**

*A>B*: 17038 > 45033 (Protein import > Peroxisome inheritance)

*Inference rule*: Domain knowledge (event A precedes event B; rule 4)

*Note*: Importing inheritance proteins to the peroxisome precedes peroxisome inheritance.

**Link 25**

*A>B*: 16559 > 16560 (Peroxisome elongation/Dnm1p-division > Docking on peroxisomal membrane)

*Inference rule*: Domain knowledge (event A precedes event B; rule 4)

*Note*: Dubious edge, possibly a false positive, even though peroxisome elongation precedes peroxisome fission and not the other way around.

**Link 26**

*A>B*: 16559 > 6625 (Peroxisome elongation/Dnm1p-division > Translocation into peroxisomal matrix)

*Inference rule*: Domain knowledge (event A precedes event B; rule 4)

*Note*: Dubious edge, possibly a false positive.

**Link 27**

*A>B*: 16559 > 16562 (Peroxisome elongation/Dnm1p-division > Peroxisomal receptor recycling)

*Inference rule*: Domain knowledge (event A precedes event B; rule 4)

*Note*: Dubious edge, possibly a false positive.

**Link 28**

*A>B*: 45033 > 16560 (Peroxisome inheritance > Docking on peroxisomal membrane)

*Inference rule*: Domain knowledge (event A precedes event B; rule 4)

*Note*: Dubious edge, possibly a false positive.

**Link 29**

*A>B*: 45033 > 6625 (Peroxisome inheritance > Translocation into peroxisomal matrix)

*Inference rule*: Domain knowledge (event A precedes event B; rule 4)

*Note*: Dubious edge, possibly a false positive.

**Link 30**

*A>B*: 32880 > 16559 (Regulation of protein localization > Peroxisome elongation/Dnm1p-division)

*Inference rule*: Experimental evidence (main components of A might influence main components of B; rule 2)

*Note*: The statement that manipulation of A (regulation of protein localization) affects B (Dnm1p-dependent fission of peroxisomes) is based on the experimental evidence that Pho85p-dependent phosphorylation of the peroxisomal fission factor Pex11p is required for regulating the localization of Pex11p to the peroxisomal membrane.

**Link 31**

*A>B*: 32880 > 6625 (Regulation of protein localization > Translocation into peroxisomal matrix)

*Inference rule*: Domain knowledge (main components of A might influence main components of B; rule 5)

*Note*: Pho85p-dependent phosphorylation of the translocation factor Pex10p might be required for translocation (by analogy to the case examined in link 30).

**Link 32**

*A>B*: 32880 > 266 (Regulation of protein localization > Regulation of Dnm1p)

*Inference rule*: Domain knowledge (main components of A might influence main components of B; rule 5)

*Note*: Members of FN 32880 (Pho85p and Rsp5p) establish non-directional PPI with the Dnm1p regulator Rpn11p in FN 266. However, the actual occurrence of post-translational modifications (i.e., directional enzyme-substrate interactions) has not been demonstrated yet.

**Link 33**

*A>B*: 32880 > 48856 (Regulation of protein localization > Cortical actin dynamics)

*Inference rule*: Experimental evidence (main components of A might influence main components of B; rule 2)

*Note*: The statement that manipulation of A (regulation of protein localization) affects B (cortical actin dynamics) is based on the experimental evidence that the ubiquitin ligase Rsp5p regulates the localization and function of the actin-related protein Sla1p.

**Link 34**

*A>B*: 48856 > 16559 (Cortical actin dynamics > Peroxisome elongation/Dnm1p-division)

*Inference rule*: Biological knowledge (A might influence B; rule 6)

*Note*: General biological knowledge suggests that cortical actin dynamics might facilitate membrane modifications (like those occurring in peroxisome fission).

**Link 35**

*A>B*: 48856 > 1 (Cortical actin dynamics > Mitochondrion Dnm1p-division)

*Inference rule*: Biological knowledge (A might influence B; rule 6)

*Note*: General biological knowledge suggests that cortical actin dynamics might facilitate membrane modifications (like those occurring in mitochondrial fission).

**Link 36**

*A>B*: 48856 > 1300 (Cortical actin dynamics > Cell aging)

*Inference rule*: Biological knowledge (A might influence B; rule 6)

*Note*: Edge of dubious significance.

**Link 37**

*A>B*: 48856 > 45033 (Cortical actin dynamics > Peroxisome inheritance)

*Inference rule*: Biological knowledge (A might influence B; rule 6)

*Note*: Edge of dubious significance.

**Link 38**

*A>B*: 17038 > 48856 (Protein import > Cortical actin dynamics)

*Inference rule*: Experimental evidence (main components of A might influence main components of B; rule 2)

*Note*: The statement that manipulation of A (protein import) affects B (cortical actin dynamics) is based on the experimental evidence that the Pex19p-dependent import of proteins to the peroxisomes imports proteins that, like Vps1p, may induce remodeling of cortical actin.

**Link 39**

*A>B*: 19538 > 16560 (Protein folding > Docking on peroxisomal membrane)

*Inference rule*: Biological knowledge (A might influence B; rule 6)

*Note*: General biological knowledge suggests that proteins of the chaperonin T-complex might assist folding of the proteins involved in docking.

**Link 40**

*A>B*: 19538 > 16559 (Protein folding > Peroxisome elongation/Dnm1p-division)

*Inference rule*: Biological knowledge (A might influence B; rule 6)

*Note*: General biological knowledge suggests that proteins of the chaperonin T-complex might assist folding of the proteins involved in peroxisome fission.

**Link 41**

*A>B*: 19538 > 266 (Protein folding > Regulation of Dnm1p)

*Inference rule*: Biological knowledge (A might influence B; rule 6)

*Note*: General biological knowledge suggests that proteins of the chaperonin T-complex might assist folding of the proteins involved in the regulation of peroxisomal and mitochondrial fission.

**Link 42**

*A>B*: 45046 > 19395 (PMP insertion into peroxisomal membrane > Fatty acid oxidation)

*Inference rule*: Domain knowledge (B logically implies A; rule 3)

*Note*: Pex11p, which mediates fatty acid oxidation, is a PMP that requires being inserted in the peroxisomal membrane for proper functioning.

**Link 43**

*A>B*: 17038 > 19395 (Protein import > Fatty acid oxidation)

*Inference rule*: Domain knowledge (event A precedes event B; rule 4)

*Note*: Importing fatty acid oxidation-related proteins into the peroxisome precedes fatty oxidation.

**Link 44**

*A>B*: 32880 > 19395 (Regulation of protein localization > Fatty acid oxidation)

*Inference rule*: Biological knowledge (A might influence B; rule 6)

*Note*: General biological knowledge suggests that regulation of localization of specific proteins (e.g., fatty acid oxidases) to specific locations (e.g., the peroxisomes) might affect their function.

**Link 45**

*A>B*: 5977 > 19395 (Glycogen biosynthesis inhibition > Fatty acid oxidation)

*Inference rule*: Domain knowledge (main components of A might influence main components of B; rule 5)

*Note*: Pho85p phosphorylates Fox2p and Pex11p and might thus regulate fatty acid oxidation.

**Link 46**

*A>B*: 5977 > 266 (Glycogen biosynthesis inhibition > Regulation of Dnm1p)

*Inference rule*: Domain knowledge (main components of A might influence main components of B; rule 5)

*Note*: Pho85p and Reg1p might indirectly regulate Dnm1p.
