# Supplementary material for: From networks of protein interactions to networks of functional dependencies
Source: BMC Syst Biol. 2012 May 20;6:44. doi: 10.1186/1752-0509-6-44 (PMC3434018; doi:10.1186/1752-0509-6-44)
Supplement: Additional file 5 — FN and edges of the cellular bud and cell budding PG (pdf). The file describes the FN and the edges of the cellular bud and cell budding PG (displayed in Figure 5A and Figure 5B of the main text, respectively), the physical links underlying the edges (crossing PPI and/or shared proteins), as well as their biochemical basis and biological significance [76-94]. [file 1752-0509-6-44-S5.doc]

**The cellular bud PG**

Part I of this file describes in detail the cellular bud PG, which is shown in Figure 5A of the main text. Part II reports the original labels of the individual FN and the way they may have been relabeled. Finally, Part III reports the individual edges and the criteria whereby they may have been defined as directional in the PG.

**Part I: Description of the cellular bud PG**

**1. The polarization of the mother cell**

**Link 1 35556>753 Bud site selection > Polarity establishment**

Crossing PPI: Bem1p_Rsr1p; Bem1p_Rga2p (Shared proteins: Cdc24p; Cdc42p)

**Link 1** connects the signaling (and Rsr1p-mediated) events responsible for selecting the bud site (node 35556) with the morphogenetic (and Cdc42p-mediated) events responsible for establishing polarity (node 753). The link is supported by the PPI of the adaptor protein Bem1p with the GTPase Rsr1p (Bud1p) and Cdc24p. Specifically, Rsr1p activates the GEF Cdc24p. In turn, Cdc24p activates Cdc42p, which is the major regulator of polarity. ‘Landmark proteins’ ensure the correct localization to the prospective budding site of Rsr1p (together with its regulators, i.e., the GEF Bud5p and the GAP Bud2p).

**Link 2 753>19236 Polarity establishment > Regulation of polarity-associated responses**

Crossing PPI: Bem1p_Cla4p (Shared protein: Cdc42p)

**Link 2** represents the dependence of Cla4p activation (node 19236) on Cdc42p activation (node 753) at the bud site. Cla4p, which is a member of the p21-activated kinase family, allows both entry in mitosis (by phosphorylating the Cdc28p inhibitor Swe1p, like Cdc5p does) [76] and septin assembly (by phosphorylating the Cdc3p and Cdc10p septins), as portrayed by the links 7 and 9, respectively.

**2. Coordinating cell budding with the cell cycle**

**Link 3 7154>753 Polarity coordination with cell division > Polarity establishment**

Crossing PPI: Rsr1p_Cdc42p; Rga2p_Cdc42p (Shared proteins: Bem1p; Cdc42p)

**Link 4 51231>7154 Cell cycle progression > Polarity coordination with cell division**

Crossing PPI: Cdc28p_Bem1p; Swe1p_Bem1p

**Link 5 51321>35556 Cell cycle progression > Bud site selection**

Crossing PPI: Cdc28p_Rga2p; Swe1p_Cdc24p

**Link 6 7154>19236 Polarity coordination with cell division>Regulation of polarity-associated responses**

Crossing PPI: Bem1p_Cdc42p; Bem1p_Cla4p; Rsr1p_Rga1p

**Links 3**-**6** represent a mechanism of positive regulation that the cell cycle exerts on the polarity system in G1 phase. The regulation involves the cyclin-dependent kinases Cdc28p and Pho85p, which phosphorylate (and cause functional inhibition) of Rga2p. Rga2p is a GAP that inhibits Cdc42p, thus ensuring that Cdc42p is activated [77]. Thus, Cdc28p (the homolog of mammalian Cdk1) is required not only for driving the cell cycle but also for coordinating cell surface growth with the cell cycle. Notably, Cdc28p (like Cdc5p and Swe1p) associates with the bud neck.

**Link 7 19236>51321 Regulation of polarity-associated responses > Cell cycle progression**

Crossing PPI: Cla4p_Swe1p; Cla4p_Cdc28p

**Link 7** represents the Cla4p-dependent inhibition of the Cdc28p inhibitor Swe1p that allows entry in mitosis. Swe1p (the homolog of mammalian Wee1) is a protein tyrosine kinase that phosphorylates Cdc28p. Swe1p expression changes during the cell cycle phases and, in G2 and M, Swe1p is rapidly degraded. Degradation of Swe1p requires its recruitment to the septin ring, where it is phosphorylated by Cla4p, Cdc5p and Cdc28p.

**Link 8 19236_35556 Regulation of polarity-associated responses – Bud site selection**

Crossing PPI: Cdc24p_Cla4p; Rsr1p_Rga1p (Shared protein: Cdc42p)

The non-directional **link 9** is likely a FP.

**3. Coordinating cell budding with the septin ring**

**Link 9 19326>31106 Regulation of polarity-associated responses > Septin ring organization**

Crossing PPI: Cla4p_Cdc11p; Cla4p_Cdc12p

**Link 9** shows that Cla4p triggers the organization of the septin-based ring at the neck of the bud, because Cla4p phosphorylates the septins Cdc3p and Cdc10p. The septin ring is composed of filament-forming septins (Cdc3p, Cdc10p, Cdc11p, Cdc12p) and septin-associated proteins (e.g., Bni5p and Kcc4p). Bni5p is involved in septin organization at the bud neck. Kcc4p is a protein kinase involved in the septin checkpoint, which negatively regulates Swe1p and shows structural homology to bud neck kinases Gin4p and Hsl1p.

**Link 10 31106>45860 Septin ring organization > Septin ring checkpoint**

Crossing PPI: Bni5p_Cdc11p (Shared protein: Cdc12p)

**Link 10** shows that the septin-associated protein Bni5p and the septins Cdc11p and Cdc12p connect the septin ring with the septin ring checkpoint. Specifically, when the septin ring has been assembled, Cdc11p and Cdc12p relieve the auto-inhibition of the Hsl1p kinase, which is a key regulator of the cell cycle [78].

**Link 11 45860>51321 Septin ring checkpoint > Cell cycle progression**

Crossing PPI: Cdc11p_Cdc28p; Cdc12p_Cdc5p

**Link 12 31106>51321 Septin ring organization > Cell cycle progression**

Crossing PPI: Kcc4p_Swe1p; Cdc3p_Cdc28p; Cdc12p_Cdc5p

**Link 11** shows that the septin ring checkpoint controls cell cycle progression. As mentioned above, a possible mechanism is the septin-dependent relief of the auto-inhibition of Hsl1p. **Link 12** shows that the septin ring-associated Kcc4p protein positively controls the cell cycle by inhibiting Swe1p.

**4. Coordinating cell budding with spindle assembly**

**Link 13 19236>51300 Regulation of polarity-associated responses > SPB and actin organization**

Crossing PPI: Cla4p_Swe1p; Rga1p_Cmd1p

**Link 14 51300>42254 SPB and actin organization > Spindle assembly checkpoint**

Crossing PPI: Cmd1p_Glc7p; Swe1p_Glc7p

**Link 15 42254>51321 Spindle assembly checkpoint > Cell cycle progression**

Crossing PPI: Glc7p_Cdc5p; Glc7p_Swe1p

**Link 13** shows that budding results in the activation of Cmd1p (calmodulin). In turn, Cmd1p exerts two major actions. First, it regulates the formation of the spindle pole body (SPB), by recruiting essential SPB components, as represented by **link 14**. Then, the SPB connects to a checkpoint that, as **link 15** shows, regulates the cell cycle. Second, Cmd1p also regulates actin assembly (see below, link 16). These links are based on Glc7p, the catalytic subunit of a serine/threonine protein phosphatase that regulates many functions, including mitosis and cell wall integrity [79].

**5. Coordinating cell budding with actin assembly**

**Link 16 51300>915 SPB and actin organization > Actin assembly at the ring & Regulation of secretion**

Crossing PPI: Cmd1p_Mlc1p; Swe1p_Mlc1p

**Link 16** shows that calmodulin, possibly through its interaction with Myo2p also regulates polarized secretion towards the bud. Ca-independent targets of Cmd1p play essential roles in SPB and actin organization, as well as budding [80]. In fungi, the SPB is a centrosome-like microtubule-organizing centre. Cmd1p is required for correct localization of the essential Spc110p component to the SPB. In addition, through its interaction with Myo2p, Cmd1p is required for polarized growth and vacuole inheritance by daughter cells.

**Link17 915>32940 Actin assembly at the ring & Regulation of secretion > Secretion to the bud along actin cables**

Crossing PPI: Mlc1p_Act1p; Myo1p_Smy1p

**Link 17** shows that, possibly via interactions with actin at the ring of the bud neck, the class II myosin chains Myo1p and Mlc1p localize to the bud and, by virtue of associations with trafficking regulators (Sec4p) and effectors (Myo2p), regulate vesicle targeting during cytokinesis [81]. Notably, Smy1p and Myo2p interact and localize to the bud [82]. Mlc1p an essential light chain not only for Myo2p but also for the class II myosin heavy chain Myo1p, which (as part of the actomyosin ring) plays a critical role in cytokinesis. Early in the cell cycle, Myo1p localizes to a ring at the presumptive bud site and remains at the mother-bud neck until cytokinesis is completed. Late in anaphase, Act1p also accumulates in the neck ring in a Myo1p-dependent manner. At the end of anaphase, the actomyosin ring decreases in size, presumably due to contraction.

**Link 18 51016>32940 Polarisome-dependent polymerization of actin > Secretion to the bud along action cables**

Crossing PPI: Bni1p_Act1p; Bnr1p_Act1p

**Link 18** represents the polarisome-induced and formin-dependent polarized assembly of actin cables along the mother-bud axis. Bni1p and Bnr1p are formins, which assemble linear actin cables in the bud (Bni1p) and bud neck (Bnr1p). Together with Spa2p, Bud6p, Pea2p, Msb3p and Msb4p, Bni1p is a component of the polarisome complex.

**Link 19 8154>32940 Actin bundle assembly > Secretion to the bud along action cables**

Crossing PPI: Tpm1p_Act1p

**Link 19** shows the stabilization of the actin cables. Tpm1p and Tpm2p are tropomyosin isoforms, which bind to and stabilize actin filaments, thereby directing polarized transport [83].

**Link 20 35556>30242 Bud site selection > Maintenance of cell wall integrity**

Crossing PPI: Rom2p_Slt2p; Rgd2p_Mkk1p

**Link 20** represents the Rom2p-dependent activation of the PKC pathway regulating cell wall integrity. Rom2p is a GEF for Rho GTPases. Specifically, Pkc1p activates the MAPKKK Bck1p, which activates the redundant MAPKK Mkk1p and Mkk2p. In turn, the MAPKK activate the MAPK Slt2p. Strains disrupted for any of these protein kinases lose osmotic stability, suggesting a lack of cell wall integrity. Notably, membrane bound Spa2p is sufficient to bind and recruit Mkk1p and Slt2p to the cell cortex, at sites of polarized growth [84].

**6. Other links**

**Link 21 6261>45860 Regulation of SPB separation and spindle alignment > Septin ring checkpoint**

Crossing PPI: Cdc28p_Cdc11p; Cdc5p_Cdc12p

**Link 21** represents a possible interplay between the checkpoints that control spindle orientation and septin assembly.

**Link 22 6261>51321 Regulation of SPB separation and spindle alignment > Cell cycle progression**

Crossing PPI: none (Shared proteins: Cdc28p; Cdc5p)

**Link 22** represents a possible effect of the spindle regulation on the progression of the cell cycle. Specifically, Cdc28p and the polo kinase Cdc5p might positively regulate SPB separation in mitosis. The spindle aligns along the mother-bud axis through interactions between cytoplasmic microtubules and the bud cortex, which is mostly mediated by Kar9p. Kar9p binds to SPB (in G1 phase), then is phosphorylated by Cdc28p (in G1/S) and finally, together with Cdc28p, moves from the SPB to the plus end of the microtubules towards the bud. Thus, the Cdc28p-regulated localization of Kar9p contributes to spindle alignment [85]. Also, Cdc5p contributes to SPB separation [86].

**Link 23 6261_7154 Regulation of SPB separation and spindle alignment – Polarity coordination with cell division**

Crossing PPI: Cdc28p_Bem1p; Cdc28p_Rga2p

The non-directional **link 23** is likely a FP.

**Link 24 6310>51321 DNA recombination > Cell cycle progression**

Crossing PPI: Cdc5p_Irc8p (Shared protein: Cdc28p)

**Link 24** might represent the effect of DNA recombination events on the cell cycle, which involve Irc8p, a protein of unknown function, which localizes to the bud tip. Its expression is regulated by the cell cycle [87].

**Link 25 6310_7154 DNA recombination – Polarity coordination with cell division**

Crossing PPI: Cdc28p_Bem1p; Cdc28p_Rga2p

The non-directional **link 25** is likely a FP.

**Link 26 51231_753 Cell cycle progression – Polarity establishment**

The non-directional **link 26** is likely a FP.

**Part II: List of FN in the cellular bud PG**

**FN 35556**

*Annotated GO term(s)*: 35556

*Original label*: Intracellular signal transduction

*New label*: Bud site selection

*Protein content*: Cdc24p, Cdc42p, Rga2p, Rgd2p, Rom2p, Rsr1p

*Notes*: FN 35556 has been labeled ‘Bud site selection’, because the node provides a minor coverage of the *S. cerevisiae* proteins annotated in the GO database by the GO:003556 term ‘Intracellular signal transduction’ (6 out of 165 proteins). The new label maintains consistency with and specifies the initial GO-based label, because the (GTPase-related signaling) events (mediated by the protein content of the FN) bring about the selection of the site of polarized growth.

**FN 753**

*Annotated GO term(s)*: 753

*Original label*: Cell morphogenesis involved in conjugation with cellular fusion

*New label*: Establishment of polarity

*Protein content*: Bem1p, Cdc24p, Cdc42p

*Notes*: FN 753 has been labeled ‘Establishment of polarity’, because the new label retains the reference of the original label GO:0000753 to morphogenetic changes in cell shape and size, but specifies that such changes occur in the context of cell budding and not cell mating (like the other FN). Thus, the new label represents more appropriately the actual relation of the FN within the PG.

**FN 19236**

*Annotated GO term(s)*: 19236

*Original label*: Response to pheromone

*New label*: Regulation of polarity-associated responses

*Protein content*: Cdc42p, Cla4p, Rga1p

*Notes*: FN 19236 has been labeled ‘Regulation of polarity-associated responses’, because the new label represents more appropriately the (Cla4p-dependent) relations of the FN with other polarity-associated functions in the PG (e.g., actin organization, septin assembly and cell cycle progression).

**FN 7154**

*Annotated GO term(s)*: 7154

*Original label*: Cell communication

*New label*: Polarity coordination with cell division

*Protein content*: Bem1p, Cdc24p, Rga2p, Rsr1p

*Notes*: FN 7154 has been labeled ‘Polarity coordination with cell division’, because the new label represents more appropriately the role of its protein content (in particular, the GAP Rga2p) in the positive regulation that the cell cycle (in particular, the cyclin-dependent kinases Cdc28p and Pho85p) exerts in G1 on budding (by inhibiting the Cdc42p inhibitor Rga2p).

**FN 51321**

*Annotated GO term(s)*: 51321

*Original label*: Meiotic cell cycle

*New label*: Cell cycle progression

*Protein content*: Cdc28p, Cdc5p, Swe1p

*Notes*: FN 51321 has been labeled ‘Cell cycle progression’, because the new label represents more appropriately the role of its protein content as regulator/target of the cell cycle/cell budding bi-directional interplay, and in particular the Cdc28p-dependent activation (via Rga2p) of Cdc42p in G1 phase and the Cla4p-dependent activation (via Swe1p) of Cdc28p in M phase.

**FN 31106**

*Annotated GO term(s)*: 31106+ 921

*Original label*: Septin ring organization + septin ring assembly

*New label*: Septin ring assembly

*Protein content*: Bni5p, Cdc12p, Cdc3p, Kcc4p

*Notes*: The node label highlights the formation of the ring-like structure at the cell cortex.

**FN 45860**

*Annotated GO term(s)*: 45860

*Original label*: Positive regulation of protein kinase activity

*New label*: Septin ring checkpoint

*Protein content*: Cdc11p, Cdc12p

*Notes*: FN 45860 has been labeled ‘Septin ring checkpoint’, because the new label represents more appropriately the role of its protein content (in particular, the septins Cdc11p and Cdc12p) as checkpoint for detecting the assembly of the septin ring and consequently activating cell cycle regulators (specifically, the kinase Hsl1p).

**FN 51300**

*Annotated GO term(s)*: 51300

*Original label*: Spindle pole body organization

*New label*: Spindle pole body and actin organization

*Protein content*: Cmd1p, Swe1p

*Notes*: In addition to the original (GO-based) term ‘Spindle pole body organization’, also the term ‘and actin organization’ has been added to FN 51300, to signify that (in the context of the cell bud), the protein content (specifically, calmodulin Cmd1p) regulates both SPB and actin, thus more appropriately representing the relation of FN 51300 with the other nodes in the graph.

**FN 42254**

*Annotated GO term(s)*: 42254

*Original label*: Ribosome biogenesis

*New label*: Spindle assembly checkpoint

*Protein content*: Glc7p, Hrr25p

*Notes*: FN 42254 has been labeled ‘Spindle assembly checkpoint’, because the new label represents more appropriately the role of its protein content (in particular, Glc7p) in the spindle assembly checkpoint that delays mitosis until the spindle is correctly assembled.

**FN 915**

*Annotated GO term(s)*: 915

*Original label*: Cytokinesis, actomyosin contractile ring assembly

*New label*: Actin assembly at the ring and Regulation of secretion

*Protein content*: Mlc1p, Myo1p

*Notes*: The label of FN 915 has been slightly changed to ‘Actin assembly at the ring’ and the notation ‘Regulation of secretion’ has been added, to more appropriately represent the role of the node content (i.e., the class II myosin chains Myo1p and Mlc1p) in regulating vesicle targeting during cytokinesis.

**FN 32940**

*Annotated GO term(s)*: 32940- 8154 - 15031

*Original label*: Secretion by cell -[Actin polymerization or depolymerization] -[Protein transport -[Vesicle transport along actin filament]]

*New label*: Secretion to the bud along actin filaments

*Protein content*: Act1p, Smy1p

*Notes*: FN 32940 has been labeled ‘Secretion to the bud along actin filaments’, because the new label represents more appropriately the role of its protein content (the actin Act1p and the Myo2p-associated protein Smy1p, both of which localize to the bud) in mediating transport of secretory vesicles towards the bud.

**FN 51016**

*Annotated GO term(s)*: 51016

*Original label*: Barbed-end actin filament capping

*New label*: Polarisome-dependent polymerization of actin

*Protein content*: Bni1p, Bnr1p

*Notes*: FN 51016 has been labeled ‘Polarisome-dependent polymerization of actin’, because the label represents more appropriately the role of its protein content (i.e., the formins Bni1p and Bnr1p) in assembling linear actin cables at the bud and the bud neck. In addition, the label specifies that the process is induced by the polarisome complex (Bnr1p being a member of the complex).

**FN 8154**

*Annotated GO term(s)*: 8154

*Original label*: Actin polymerization or depolymerization

*New label*: Actin bundle assembly

*Protein content*: Tpm1p, Tpm2p

*Notes*: FN 8154 has been slightly relabeled ‘Actin filament bundle assembly’, to specify the role of its protein content (i.e., the tropomyosin isoforms Tpm1p and Tpm2p) in binding and stabilizing actin filaments and in bundling together in actin cables.

**FN 30242**

*Annotated GO term(s)*: 30242

*Original label*: Peroxisome degradation

*New label*: Maintenance of cell wall integrity

*Protein content*: Mkk1p, Slt2p

*Notes*: FN 30242 has been labeled ‘Maintenance of cell wall integrity’, to represent the role of its protein content (i.e., the MAPKK Mkk1p and its target, the MAPK Slt2p) in regulating osmotic stability (by maintaining cell wall integrity), upon activation of the PKC pathway.

**FN 6261**

*Annotated GO term(s)*: 6261+ 10696

*Original label*: DNA-dependent DNA replication + Positive regulation of spindle pole body separation

*New label*: Regulation of spindle pole body separation and spindle alignment

*Protein content*: Cdc28p, Cdc5p

*Notes*: The node label has been slightly changed.

**FN 6310**

*Annotated GO term(s)*: 6310

*Original label*: DNA recombination

*New label*: Mitotic recombination

*Protein content*: Cdc28p, Irc8p

*Notes*: The node label has been slightly changed.

**Part III: Inference of edge direction in the cellular bud PG**

**Link 1**

*A > B*: 35556 > 753 (Bud site selection > Polarity establishment)

*Inference rule:* Domain knowledge (event A precedes event B; rule 4)

*Note:* Selecting the site of budding precedes the polarized growth of the bud site that occurs during the establishment of polarity.

**Link 2**

*A > B*: 753 > 19236 (Polarity establishment > Regulation of polarity-associated responses)

*Inference rule:* Experimental evidence (the main component of A might influence the main component of B; rule 2)

*Note:* The statement that manipulation of A (establishment of polarity) affects B (regulation of polarity-associated responses) is based on the experimental evidence that Cdc42p activation at the bud site (node 753) is required for activating the Cla4p kinase (node 19236) at the bud site.

**Link 3**

*A > B*: 7154 > 753 (Polarity coordination with cell division > Polarity establishment)

**Link 4**

*A > B*: 51231 > 7154 (Cell cycle progression > Polarity coordination with cell division)

**Link 5**

*A > B*: 51321 > 35556 (Cell cycle progression > Bud site selection)

**Link 6**

*A > B*: 7154 > 19236 (Polarity coordination with cell division > Regulation of polarity-associated responses)

*Inference rule:* Experimental evidence (the main component of A might influence the main component of B; rule 2)

*Note:* The statement that manipulation of A (polarity coordination with cell division) affects B (polarity establishment) is based on the experimental evidence that cyclin-dependent inhibition of the Cdc42p inhibitor Rga2p is required for activating Cdc42p in G1 phase.

**Link 7**

*A > B*: 19236 > 51321 (Regulation of polarity-associated responses > Cell cycle progression)

*Inference rule:* Experimental evidence (the main component of A might influence the main component of B; rule 2)

*Note:* The statement that manipulation of A (regulation of polarity-associated responses) affects B (cell cycle progression) is based on the experimental evidence that Cla4p-dependent inhibition of the Cdc42p inhibitor Swe1p is required for activating Cdc28p in M phase.

**Link 8**

*A > B*: 19236_35556 (Regulation of polarity-associated responses – Bud site selection)

*Inference rule:* Biological knowledge (A might influence B; rule 6)

*Note:* Edge of dubious significance.

**Link 9**

*A > B*: 19326 > 31106 (Regulation of polarity-associated responses > Septin ring organization)

*Inference rule:* Domain knowledge (the main component of A might influence the main component of B; rule 5)

*Note:* Cla4p-dependent phosphorylation of the septins Cdc3p and Cdc10p might be required for organizing septins into the septin ring.

**Link 10**

*A > B*: 31106 > 45860 (Septin ring organization > Septin ring checkpoint)

*Inference rule:* Domain knowledge (event A precedes event B; rule 4)

*Note:* Organizing the septin ring precedes the assembly of the septin ring-based checkpoint.

**Link 11**

*A > B*: 45860 > 51321 (Septin ring checkpoint > Cell cycle progression)

*Inference rule:* Experimental evidence (the main component of A might influence the main component of B; rule 2)

*Note:* The statement that manipulation of A (septin ring checkpoint) affects B (cell cycle progression) is based on the experimental evidence that, following the organization of the septin checkpoint, the septins Cdc11p and Cdc12p relieve the auto-inhibition of the Hsl1p kinase, a regulator of cell cycle progression.

**Link 12**

*A > B*: 31106 > 51321 (Septin ring organization > Cell cycle progression)

*Inference rule:* Experimental evidence (the main component of A might influence the main component of B; rule 2)

*Note:* The statement that manipulation of A (septin ring organization) affects B (cell cycle progression) is based on the experimental evidence that the septin-associated Kcc4p inhibits Swe1p, a negative regulator of Cdc28p.

**Link 13**

*A > B*: 19236 > 51300 (Regulation of polarity-associated responses > SPB and actin organization)

*Inference rule:* Domain knowledge (event A precedes event B; rule 4)

*Note:* Activating regulators of polarity-associated responses precedes two of such responses (i.e., the organization of both SPB and actin).

**Link 14**

*A > B*: 51300 > 42254 (SPB and actin organization > Spindle assembly checkpoint)

*Inference rule:* Domain knowledge (event A precedes event B; rule 4)

*Note:* Organization of the spindle precedes the activation of the spindle-based checkpoint.

**Link 15**

*A > B*: 42254 > 51321 (Spindle assembly checkpoint > Cell cycle progression)

*Inference rule:* Biological knowledge (A might influence B; rule 6)

*Note:* General biological knowledge suggests that cell cycle checkpoints (like the spindle assembly checkpoint discussed here) might influence the progression of the cell cycle.

**Link 16**

*A > B*: 51300 > 915 (SPB and actin organization > Actin assembly at the ring & Regulation of secretion)

*Inference rule:* Biological knowledge (A might influence B; rule 6)

*Note:* General biological knowledge suggests that actin organization is required for assembling actin cables that in general may serve many functions (including vesicle secretion).

**Link17**

*A > B*: 915 > 32940 (Actin assembly at the ring & Regulation of secretion > Secretion to the bud along actin cables)

*Inference rule:* Biological knowledge (A might influence B; rule 6)

*Note:* General biological knowledge suggests that actin assembly into cables might be required for supporting secretion of vesicles along the cables (in this case, towards the bud).

**Link 18**

*A > B*: 51016 > 32940 (Polarisome-dependent polymerization of actin > Secretion to the bud along action cables)

*Inference rule:* Domain knowledge (the main component of A might influence the main component of B; rule 5)

*Note:* Formation of the polarisome complex is known to induce the formation of actin cables, along which polarized secretion (i.e., directional, in this case towards the bud) may occur.

**Link 19**

*A > B*: 8154 > 32940 (Actin bundle assembly > Secretion to the bud along action cables)

*Inference rule:* Domain knowledge (event A precedes event B; rule 4)

*Note:* Formation of actin cables precedes the secretion of vesicles along the cables.

**Link 20**

*A > B*: 35556 > 30242 (Bud site selection > Maintenance of cell wall integrity)

*Inference rule:* Biological knowledge (A might influence B; rule 6)

*Note:* General biological knowledge suggests that activation of PKC signaling (for instance, in response to the small G protein Rom2p) might trigger (among other responses) the (MAP kinase-dependent) cell wall integrity pathway.

**Link 21**

*A > B*: 6261 > 45860 (Regulation of SPB separation and spindle alignment > Septin ring checkpoint)

*Inference rule:* Biological knowledge (A might influence B; rule 6)

*Note:* The edge is difficult to interpret in terms of directionality. However, general biological knowledge suggests that spindle dynamics (more in general, cell cycle-associate responses) might be required for regulating the polarity-associated checkpoints.

**Link 22**

*A > B*: 6261 > 51321 (Regulation of SPB separation and spindle alignment > Cell cycle progression)

*Inference rule:* Biological knowledge (A might influence B; rule 6)

*Note:* General biological knowledge suggests that spindle dynamics might contribute to the progression of the cell cycle.

**Link 23**

*A > B*: 6261_7154 (Regulation of SPB separation and spindle alignment – Polarity coordination with cell division)

*Inference rule:* The edge has been interpreted as non-directional.

*Note:* Edge of dubious significance, possibly a false positive.

**Link 24**

*A > B*: 6310 > 51321 (DNA recombination > Cell cycle progression)

*Inference rule:* knowledge (A might influence B; rule 6)

*Note:* Edge of dubious significance.

**Link 25**

*A > B*: 6310_7154 (DNA recombination – Polarity coordination with cell division)

*Inference rule:* The edge has been interpreted as non-directional.

*Note:* Edge of dubious significance, possibly a false positive.

**Link 26**

*A > B*: 51231_753 (Cell cycle progression – Polarity establishment)

*Inference rule:* The edge has been interpreted as non-directional.

*Note:* Edge of dubious significance, possibly a false positive.

**The cell budding PG**

Part I of this file describes in detail the cell budding PG, which is shown in Figure 5B of the main text. Part II reports the original labels of the individual FN and the way they may have been relabeled. Finally, Part III reports the individual edges and the criteria whereby they may have been defined as directional in the PG.

**Part I: Description of the cell budding PG**

**1. Polarized secretion**

**Link 1 753>750 = Positive regulation of polarity establishment > Polarity establishment**

Crossing PPI: Cdc24p_Cdc42p

**Link 2 750>19236 = Polarity establishment > Regulation of polarity-associated responses**

Crossing PPI: Cdc42p_Cla4p

**Link 1** represents the interaction between the Guanine nucleotide Exchange Factor Cdc24p (node 753) and its target Cdc42p, a small GTPase that is essential for establishing cell polarity (node 750). Furthermore, **link 2** represents the Cdc42p-dependent activation of the kinase Cla4p (node 19236), which regulates many budding-related responses.

**Link 3 750>6887 = Polarity establishment > Exocyst-dependent exocytosis**

Crossing PPI: Cdc42p_Exo70p

**Link 3** represents that the activation of Cdc42p (node 750) stimulates the exocyst-mediated secretion of post-Golgi vesicles towards the budding site (node 6887). The exocyst is a protein complex (comprising Exo70p, Exo84p, Sec3p, Sec5p, Sec6p, Sec8p, Sec10p and Sec15p) that associates with the plasma membrane to determine where vesicles dock and fuse. Exo70p is the direct effector of Cdc42p, based on both physical and genetic interactions. For instance, gain-of-function EXO70 mutants suppress the defective exocytic function of CDC42 mutants [88].

**Link 4 6887>132 = Exocyst-dependent exocytosis > Transport of vesicles and organelles**

Crossing PPI: Sec10p_Act1p

**Link 5 7107>132 = Membrane fusion of post-Golgi vesicles > Transport of vesicles and organelles**

Crossing PPI: Sec4p_Myo2p

The polarized transport of vesicles to the bud (node 132) depends both on (**link 4**) the exocyst-mediated docking of secretory vesicles (node 6887) and on (**link 5**) their fusion with the plasma membrane (node 7107). In particular, link 5 represents the Myo2p-dependent recruitment of the Rab GTPase Sec4p [89].

**2. Polarized assembly of actin**

**Link 6 31384>7569 = Polarisome assembly at the bud site > Actin nucleation at the bud**

Crossing PPI: Pea2p_Bud6p; Spa2p_Bud6p

Pea2p, Spa2p and Bud6p (together with Msb3p, Msb4p and Bni1p) are components of the polarisome complex, which acts as the nucleation point for polymerizing actin monomers at the bud. **Link 6** represents the link between the polarisome (node 31384) and the actin nucleation site that is localized to the bud (node 7569). In particular, Bud6p, which is an actin- and formin-interacting protein, stimulates actin nucleation by recruiting actin monomers to the formin Bni1p.

**Link 7 7569>8154 = Actin nucleation at the bud > Actin bundle assembly**

Crossing PPI: Act1p_Tpm1p; Act1p_Tpm2p

**Link 8 7569>10324 = Actin nucleation at the bud > Actin cortical patch assembly**

Crossing PPI: Act1p_Las17p

Following actin nucleation at the bud (node 7569), two major changes in the actin cytoskeleton ensue, which are represented by **link 7** and **link 8**, i.e., the assembly of actin bundles (node 8154) and the cortical actin patch (node 10324), respectively.

**Link 9 8154>132 = Actin bundle assembly > Transport of vesicles and organelles**

Crossing PPI: Tpm1p_Act1p; Tpm2p_Act1p

**Link 10 10324>132 = Actin cortical patch assembly > Transport of vesicles and organelles**

Crossing PPI: Las17p_Act1p

**Link 11 7569>132 = Actin nucleation at the bud > Transport of vesicles and organelles**

Crossing PPI: Bud6p_Myo2p (Shared protein: Act1p)

**Link 9** and **link 10** represent the dependence of the actomyosin-based transport (node 132) on the assembly of both types of actin structures (cables and patches, respectively). Specifically, node 8154 represents the stabilization of actin filaments into bundles of actin cables, which is mediated by the tropomyosins Tpm1p and Tpm2p [83]. Node 10324 represents the activation of the Arp2/3 protein complex, which depends on the actin assembly factor Las17p for nucleating branched filaments of actin. Actually, Las17p localizes with the Arp2/3 complex to actin patches. In addition, LAS17 disruption affects the Las17p- Arp2/3 co-localization and causes loss of the patches [90]. In addition, node 10324 represents the polarity-related activation of the class I myosins Myo3p and Myo5p at the patch [91]. Finally, **link 11** is likely a false positive.

**Link 12 147>10324 = Regulation of cortical actin > Actin cortical patch assembly**

Crossing PPI: End3p_Las17p; End3p_Myo3p

**Link 12** shows that the regulation of the cortical actin patch (node 10324) also depends on additional molecular mechanisms (node 147), in particular on the cortical actin and endocytosis regulators End3p and Pan1p [92].

**Link 13 747>10324 = Bud emergence > Actin cortical patch assembly**

Crossing PPI: Cmd1p_Las17p; Cmd1p_Myo3p

**Link 14 32505>747 = Membrane fusion > Bud emergence**

Crossing PPI: Glc7p_ Cmd1p

**Link 13** represents the dependence of the assembly of the cortical patch (node 10324) on the emergence of the bud (node 747), while **link 14** represents the dependence of bud emergence on membrane fusion events (node 32505). Specifically, both bud emergence and membrane fusion events refer to involvement in cell budding of calmodulin Cmd1p (node 747) [80, 93] and Glc7p, which is the catalytic subunit of a serine/threonine protein phosphatase (node 32505) [79, 94].

**Link 15 6887_7569 = Exocyst-dependent exocytosis – Actin nucleation at the bud**

Crossing PPI: Sec10p_Act1p

The non-directional **link 15** is possibly a false positive.

**Part II: List of FN in the cell budding PG**

**FN 753**

*Annotated GO term(s)*: 753-750

*Original label*: Cell morphogenesis involved in conjugation with cellular fusion -[Pheromone-dependent signal transduction involved in conjugation with cellular fusion]

*New label*: Positive regulation of polarity establishment

*Protein content*: Cdc24p

*Notes*: FN 753 has been labeled ‘Positive regulation of polarity establishment’, because the new label more specifically describes the functional role of its protein content (the GEF Cdc24p) and its relation with the neighboring node 750 (the activation of Cdc42p).

**FN 750**

*Annotated GO term(s)*: 750+749

*Original label*: Pheromone-dependent signal transduction involved in conjugation with cellular fusion + Response to pheromone involved in conjugation with cellular fusion

*New label*: Polarity establishment

*Protein content*: Cdc42p

*Notes*: FN 750 has been labeled ‘Polarity establishment’, because the new label more specifically describes the functional role of its protein content (the key polarity regulator Cdc42p).

**FN 19236**

*Annotated GO term(s)*: 19236

*Original label*: Response to pheromone

*New label*: Regulation of polarity-associated responses

*Protein content*: Cla4p

*Notes*: FN 19236 has been labeled ‘Regulation of polarity-associated responses’, because the new label more specifically describes the functional role of its protein content (i.e., the Cla4p kinase, which controls many responses during cell budding, as detailed in the cellular bud PG).

**FN 6887**

*Annotated GO term(s)*: 6887

*Original label*: Exocytosis

*New label*: Exocyst-dependent exocytosis

*Protein content*: Exo70p, Sec10p

*Notes*: FN 6887 has been labeled ‘Exocyst-dependent exocytosis’, to specify the function of its protein content and its relation with FN 750. Specifically, both Exo70p and Sec10p are members of the exocyst complex (and Exo70p is the direct target of Cdc42p).

**FN 132**

*Annotated GO term(s)*: 132+ 30050 + 32506

*Original label*: Establishment of mitotic spindle orientation + Vesicle transport along actin filament + Cytokinetic process

*New label*: Transport of vesicles and organelles

*Protein content*: Act1p, Myo2p

*Notes*: FN 132 has been labeled ‘Transport of vesicles and organelles’, because it describes more appropriately the role of its protein content in the functional context of the domain.

**FN 7107**

*Annotated GO term(s)*: 7107

*Original label*: Membrane addition at site of cytokinesis

*New label*: Membrane fusion of post-Golgi vesicles

*Protein content*: Sec4p

*Notes*: FN 7107 has been labeled ‘Membrane fusion of post-Golgi vesicles’, because it represents more appropriately the role of its protein content (the Rab GTPase Sec4p) in exocytosis.

**FN 31384**

*Annotated GO term(s)*: 31384+ 31383 + 31385

*Original label*: Regulation of initiation of mating projection growth + Regulation of termination of mating projection growth.

*New label*: Polarisome assembly at the bud site

*Protein content*: Pea2p, Spa2p

*Notes*: FN 31384 has been labeled ‘Polarisome assembly at the bud site’ to specify the function of its protein content and its relation with FN 7569. Specifically, Pea2p and Spa2p (in node 31384) are both members of the polarisome complex and are linked to Bud6p (in node 7569), which is another member of the complex.

**FN 7569**

*Annotated GO term(s)*: 7569

*Original label*: Cell aging

*New label*: Actin nucleation at the bud

*Protein content*: Act1p, Bud6p

*Notes*: FN 7569 has been labeled ‘Actin nucleation at the bud’, because the new label is more appropriate to specify the functional role of its protein content and its relations with the other FN, both upstream (the polarisome-dependent activation of actin nucleation portrayed by FN 31384) and downstream (the assembly of actin cables and patches, in FN 8154 and 10324, respectively).

**FN 8154**

*Annotated GO term(s)*: 8154+ 8298 + 51017

*Original label*: Actin polymerization or depolymerization + Intracellular mRNA localization + Actin filament bundle assembly

*New label*: Actin bundle assembly

*Protein content*: Tpm1p, Tpm2p

*Notes*: FN 8154 has been slightly relabeled ‘Actin bundle assembly’, to specify the role of its protein content (i.e., the tropomyosins Tpm1p and Tpm2p) in bundling actin filaments within actin cables.

**FN 10324**

*Annotated GO term(s)*: 10324- 48610

*Original label*: Membrane invagination -[Cellular process involved in reproduction]

*New label*: Actin cortical patch assembly

*Protein content*: Las17p, Myo3p

*Notes*: FN 10324 has been labeled ‘Actin cortical patch assembly’, because the new label is more appropriate to specify the functional role of its protein content. In particular, Las17p (the activator of the Arp2/3 complex) and Myo3p (a class I myosin) both localize to the cortical patches.

**FN 147**

*Annotated GO term(s)*: 147

*Original label*: Actin cortical patch assembly

*New label*: Regulation of cortical actin

*Protein content*: End3p, Pan1p

*Notes*: The label of FN 147 has been slightly changed to ‘Regulation of cortical actin’, to better specify the role of its functional relation with the downstream FN 10324.

**FN 747**

*Annotated GO term(s)*: 747

*Original label*: Conjugation with cellular fusion

*New label*: Bud emergence

*Protein content*: Cmd1p

*Notes*: FN 747 has been labeled ‘Bud emergence’, because the new label more appropriately describes the functional role of its protein content (calmodulin) in the initial phase of cell budding.

**FN 32505**

*Annotated GO term(s)*: 32505- 747

*Original label*: Reproduction of a single-celled organism -[Conjugation with cellular fusion]

*New label*: Membrane fusion

*Protein content*: Glc7p

*Notes*: FN 32505 has been labeled ‘Membrane fusion’, because the new label more appropriately describes the functional role of its protein content in the initial phase of cell budding.

**Part III: Inference of edge direction in the cell budding PG**

**Link 1**

*A>B*: 753>750 (Positive regulation of polarity establishment > Polarity establishment)

*Inference rule*: Experimental evidence (the main component of A might influence the main component of B; rule 2)

*Note*: The statement that manipulation of A (Positive regulation of polarity establishment) affects B (Polarity establishment) is based on the experimental evidence that Cdc24p-dependent activation of the key polarity regulator factor Cdc42p is required for activating the polarity system at the correct location in the mother cell.

**Link 2**

*A>B*: 750>19236 (Polarity establishment > Regulation of polarity-associated responses)

*Inference rule*: Experimental evidence (the main component of A might influence the main component of B; rule 2)

*Note*: The statement that manipulation of A (Polarity establishment) affects B (Regulation of polarity-associated responses) is based on the experimental evidence that Cdc42p-dependent activation of the Cla4p kinase is required for activating polarity-associated responses.

**Link 3**

*A>B*: 750>6887 (Polarity establishment > Exocyst-dependent exocytosis)

*Inference rule*: Experimental evidence (the main component of A might influence the main component of B; rule 2)

*Note*: The statement that manipulation of A (Polarity establishment) affects B (Exocyst-dependent exocytosis) is based on the experimental evidence that Cdc42p-dependent activation of the Exo70p exocyst component is required for activating exocytosis towards the correct localization of the bud.

**Link 4**

*A>B*: 6887>132 (Exocyst-dependent exocytosis > Transport of vesicles and organelles)

*Inference rule*: Domain knowledge (event A precedes event B; rule 4)

*Note*: Exocyst-dependent exocytosis precedes the transport of secretory vesicles

**Link 5**

*A>B*: 7107>132 (Membrane fusion of post-Golgi vesicles > Transport of vesicles and organelles)

*Inference rule*: Biological knowledge (A might influence B; rule 6)

*Note*: General biological knowledge suggests that membrane fusion of post-Golgi vesicles is required for the efficient transport of vesicles.

**Link 6**

*A>B*: 31384>7569 (Polarisome assembly at the bud site > Actin nucleation at the bud)

*Inference rule*: Experimental evidence (the main component of A might influence the main component of B; rule 2)

*Note*: The statement that manipulation of A (Polarity establishment) affects B (Exocyst-dependent exocytosis) is based on the experimental evidence that Cdc42p-dependent activation of the Exo70p exocyst component is required for activating exocytosis towards the correct localization of the bud.

**Link 7**

*A>B*: 7569>8154 (Actin nucleation at the bud > Actin bundle assembly)

*Inference rule*: Domain knowledge (event A precedes event B; rule 4)

*Note*: The localized nucleation of actin precedes its assembly into actin filaments and bundles.

**Link 8**

*A>B*: 7569>10324 (Actin nucleation at the bud > Actin cortical patch assembly)

*Inference rule*: Domain knowledge (event A precedes event B; rule 4)

*Note*: The localized nucleation of actin precedes its assembly into cortical patches.

**Link 9**

*A>B*: 8154>132 (Actin bundle assembly > Transport of vesicles and organelles)

*Inference rule*: Biological knowledge (A might influence B; rule 6)

*Note*: General biological knowledge suggests that formation of actin bundles along the mother-bud axis is required for the transport of vesicles and organelles that travel along such bundles.

**Link 10**

*A>B*: 10324>132 (Actin cortical patch assembly > Transport of vesicles and organelles)

*Inference rule*: Biological knowledge (A might influence B; rule 6)

*Note*: General biological knowledge suggests that localized formation of a cortical actin patch is required for the transport of vesicles.

**Link 11**

*A>B*: 7569>132 (Actin nucleation at the bud > Transport of vesicles and organelles)

*Inference rule*: Biological knowledge (A might influence B; rule 6)

*Note*: Edge of dubious interpretation, possibly a false positive.

**Link 12**

*A>B*: 147>10324 (Regulation of cortical actin > Actin cortical patch assembly)

*Inference rule*: Domain knowledge (event A precedes event B; rule 4)

*Note*: The regulation of cortical actin precedes its assembly into a cortical patch.

**Link 13**

*A>B*: 747>10324 (Bud emergence > Actin cortical patch assembly)

*Inference rule*: Biological knowledge (A might influence B; rule 6)

*Note*: General biological knowledge suggests that the emergence of the bud might be required for the assembly of the cortical patch of actin.

**Link 14**

*A>B*: 32505>747 (Membrane fusion > Bud emergence)

*Inference rule*: Biological knowledge (A might influence B; rule 6)

*Note*: General biological knowledge suggests that membrane dynamics might be required for the emergence of the bud.

**Link 15**

*A>B*: 6887_7569 (Exocyst-dependent exocytosis – Actin nucleation at the bud)

*Inference rule*: No directionality has been inferred for this edge.

*Note*: Edge of dubious interpretation, possibly a false positive.
