# Supplementary material for: From networks of protein interactions to networks of functional dependencies
Source: BMC Syst Biol. 2012 May 20;6:44. doi: 10.1186/1752-0509-6-44 (PMC3434018; doi:10.1186/1752-0509-6-44)
Supplement: Additional file 8 — Comparative analysis (pdf). The file describes the network analysis-based comparison of PG obtained with our method and with the method described in reference 5. [file 1752-0509-6-44-S8.doc]

**Comparative analysis**

To provide a quantitative comparison of our approach with another method, we have focused on graphs that can be obtained with the algorithm devised by Dotan-Cohen et al (reference 5 of main text). Specifically, we have compared the three PG that are discussed in our manuscript (i.e., *S. cerevisiae* cell budding, cellular bud and peroxisome) with three related graphs, which have been assembled by retrieving (from the Dotan-Cohen dataset) homologous FN (i.e., nodes with the same GO labels) and their mutual edges. Results of a network analysis-based comparison are shown in Table 1. The comparison is only in part feasible, because not all the GO terms used to define our FN are also present in the Dotan-Cohen dataset.

On one hand, the budding and bud graphs obtained with our method (compared with the Dotan-Cohen method) provide a more sparse representation, as quantified by lower <*k*> (average number of edges per node), lower network density (<*k*> normalized to a clique with the same number of edges), lower <*C*> (average clustering coefficient) and higher <*l*> (average length). On the other hand, according to the same criteria, the peroxisome graph produced with our method is less sparse.

Taken together, we propose that these findings provide interesting insights into the ability of the algorithm to establish how functions can be related, depending on the type of information that is associated with the underlying molecular domain. Specifically, to define the cell budding and cellular bud domains, we selected only proteins directly annotated with the GO terms that specifically indicate the domain. In these cases, the algorithm produces a limited number of FN, which are well characterized in terms of protein content. So, when the algorithm only deals with core proteins, the FN can map the PPI network with just a limited number of overlaps. In contrast, to define the peroxisome domain, we selected not only the core (i.e., peroxisomal) proteins but also their ‘first-degree neighbors’, which provides an example (among others) of how the algorithm behaves with non-core proteins. In this case, the first layer of neighbors remains exceedingly covered by FN, even after the FN are made to compete for including the proteins in their content (step 4 of the algorithm pseudocode). This occurs, because a large set of proteins cannot be differentiated in terms of PMS and, therefore, a large number of overlaps among FN are turned into edges (step 6 of the algorithm pseudocode). The final consequence of the two scenarios discussed here is that the former (but not the latter) type of markov representations have relations among FN that are mediated by many other FN.

Finally, as pointed out throughout the main text, the systematic validation of our graphical representations has been performed always in the light of the available biological knowledge of the represented domains. This kind of validation has highlighted false positives and false negatives in the relations among FN. Here, to provide such quantitative validation in a more concise format, we have summarized in Table 2 the results shown in the *Additional Files 4 and 5*. The false positive and negative edges are summarized in Table 3.

**Table 1.** *Network analysis-based comparison*

| **Process graph** | **Reference** | **Nodes** | **Edges** | **<*k*>** | **density** | **<*C*>** | **<*l*>** |
| --- | --- | --- | --- | --- | --- | --- | --- |
| Cell budding | This manuscript | 42 | 59 | 2,81 | 0,069 | 0,237 | 3,999 |
|  | Dotan-Cohen et al | 31 | 321 | 16,65 | 0,555 | 0,764 | 1,482 |
| Cellular bud | This manuscript | 102 | 682 | 13,37 | 0,132 | 0,360 | 2,352 |
|  | Dotan-Cohen et al | 67 | 613 | 16,03 | 0,243 | 0,574 | 2,086 |
| Peroxisome | This manuscript | 248 | 5701 | 45,98 | 0,186 | 0,597 | 1,934 |
|  | Dotan-Cohen et al | 193 | 2653 | 24,10 | 0,126 | 0,464 | 2,283 |

**Table 2.** *Biological knowledge-based validation of the PG reported in the manuscript*

| **Process Graph** | **Links** | **True Positives** | **False Positives** | **False Negatives** |
| --- | --- | --- | --- | --- |
| Cell budding | 16 | 13 (81,3%) | 2 (12,5%) | 1 (6,3%) |
| Cellular bud | 27 | 23 (85,2%) | 3 (11,1%) | 1 (3,7%) |
| Peroxisome | 48 | 38 (79,2%) | 8 (16,7%) | 2 (4,2%) |

**Table 3.** *False positives and negatives in the PG reported in the manuscript*

| **Cell budding** | **Link** | **FN A and B** |
| --- | --- | --- |
| False Positives  (2/16; 12,5%) | 11 | 7569>132 (Actin nucleation at the bud > Transport of vesicles and organelles) |
|  | 15 | 6887_7569 (Exocyst-dependent exocytosis – Actin nucleation at the bud) |
| False Negatives  (1/16; 6,3%) | missing | 750 > 7569 (Polarity establishment > Actin nucleation at the bud) |
| **Cellular bud** | **Link** | **FN A and B** |
| False Positives  (3/27; 11,1%) | 23 | 6261_7154 (Regulation of SPB separation and spindle alignment – Polarity coordination with cell division) |
|  | 24 | 6310>51321 (DNA recombination > Cell cycle progression) |
|  | 25 | 6310_7154 (DNA recombination – Polarity coordination with cell division) |
| False Negatives  (1/27; 3,7%) | missing | 19236 > 32940 (Regulation of polarity-associated responses > Secretion to the bud along action cables) |
| **Peroxisome** | **Link** | **FN A and B** |
| False Positives  (8/48; 16,7%) | 4 | 16560 > 16562 (Docking on peroxisomal membrane > Peroxisomal receptor recycling) |
|  | 25 | 16559 > 16560 (Peroxisome elongation/Dnm1p-division > Docking on peroxisomal membrane) |
|  | 26 | 16559 > 6625 (Peroxisome elongation/Dnm1p-division > Translocation into peroxisomal matrix) |
|  | 27 | 16559 > 16562 (Peroxisome elongation/Dnm1p-division > Peroxisomal receptor recycling) |
|  | 28 | 45033 > 16560 (Peroxisome inheritance > Docking on peroxisomal membrane) |
|  | 29 | 45033 > 6625 (Peroxisome inheritance > Translocation into peroxisomal matrix) |
|  | 36 | 48856 > 1300 (Cortical actin dynamics > Cell aging) |
|  | 37 | 48856 > 45033 (Cortical actin dynamics > Peroxisome inheritance) |
| False Negatives  (2/48; 4,2%) | missing | 16562 > 45184 (Peroxisomal receptor recycling > Peroxisomal receptor-enzyme recognition) |
|  | missing | 32581 > 45046 (ER-dependent peroxisome biogenesis > PMP insertion into peroxisomal membrane) |
